# Habitat Adaptation Drives Speciation of a *Streptomyces* Species with Distinct Habitats and Disparate Geographic Origins

**DOI:** 10.1128/mbio.02781-21

**Published:** 2022-01-11

**Authors:** Jiao Wang, Yisong Li, Adrián A. Pinto-Tomás, Kun Cheng, Ying Huang

**Affiliations:** a State Key Laboratory of Microbial Resources, Institute of Microbiology, Chinese Academy of Sciences, Beijing, China; b Center for Research in Cell and Molecular Biology, Center for Research in Microscopic Structures and Biochemistry Department, School of Medicine, University of Costa Rica, San José, Costa Rica; c College of Life Sciences, University of Chinese Academy of Sciences, Beijing, China; University of California, Berkeley

**Keywords:** *Streptomyces*, gene flow, habitat adaptation, lifestyle, population genetics, speciation

## Abstract

Microbial diversification is driven by geographic and ecological factors, but how the relative importance of these factors varies among species, geographic scales, and habitats remains unclear. *Streptomyces*, a genus of antibiotic-producing, spore-forming, and widespread bacteria, offers a robust model for identifying the processes underlying population differentiation. We examined the population structure of 37 Streptomyces olivaceus strains isolated from various sources, showing that they diverged into two habitat-associated (free-living and insect-associated) and geographically disparate lineages. More frequent gene flow within than between the lineages confirmed genetic isolation in *S. olivaceus*. Geographic isolation could not explain the genetic isolation; instead, habitat type was a strong predictor of genetic distance when controlling for geographic distance. The identification of habitat-specific genetic variations, including genes involved in regulation, resource use, and secondary metabolism, suggested a significant role of habitat adaptation in the diversification process. Physiological assays revealed fitness trade-offs under different environmental conditions in the two lineages. Notably, insect-associated isolates could outcompete free-living isolates in a free-iron-deficient environment. Furthermore, substrate (e.g., sialic acid and glycogen) utilization but not thermal traits differentiated the two lineages. Overall, our results argue that adaptive processes drove ecological divergence among closely related streptomycetes, eventually leading to dispersal limitation and gene flow barriers between the lineages. *S. olivaceus* may best be considered a species complex consisting of two cryptic species.

## INTRODUCTION

Delineating microbial populations is vitally important in understanding microbial evolution and diversity (1). However, the concept of microbial species and the processes contributing to microbial speciation remain controversial (2, 3). In the last decade, several operational approaches have been proposed to address this issue (4–6). These advances, combined with the rapid proliferation of genomic data, have greatly facilitated research on the processes that shape population genetic patterns.

Traditionally, microorganisms are considered cosmopolitan due to their large population sizes and high dispersal potential (7), but this view has recently been challenged (8). As in larger organisms, isolation by distance (IBD) has been reported in bacteria and archaea (9–11), which means that the genetic differentiation of strains increases with geographic distance (12). This pattern could be generated by drift or past selection and subsequently be maintained by spatial dispersal limitation (10, 13). Straightforwardly, due to restricted dissemination and thus reduced encounters, gene flow barriers could arise from geographic isolation, eventually resulting in allopatric speciation (14). Alternatively, barriers to gene flow could emerge via ecological differentiation independent of geographic distance (15, 16), which is common among microbial populations and is also known as isolation by environment (IBE) (17). Several studies have revealed that closely related strains coexisting at a small spatial scale can be clustered into ecologically and genetically divergent groups where recombination barriers exist without physical isolation (18, 19). Furthermore, given that environmental differences may be correlated with geographic distance, the effect of geographic isolation is probably overestimated (10). In summary, microbial speciation is driven by multiple interacting processes, yet it is unclear how the relative importance of each process varies among spatial scales and habitat types.

In addition, the effects of these processes might depend on the physiological traits of microbes (10). For example, bacteria of the genera *Bacillus* and *Streptomyces* can produce stress-resistant spores, which enhance their dispersal and survival. Therefore, these spore-forming microorganisms are globally distributed with little dispersal limitation among ecologically similar habitats (20–23). Their distribution patterns are governed by either environmental selection (20, 24) or historical demographic processes driven by paleoclimate dynamics (21–23). Nevertheless, previous studies analyzed microorganisms either at a relatively low genetic resolution or with a quite high level of genetic differentiation. A more interesting issue is that most published works on microbial speciation focused on populations either at local scales with distinct habitats (6, 25) or across large spatial scales with the same ecological niche (11, 23). However, many microbial species are widely distributed in terms of both habitat and geography. Further surveys concentrating on highly related strains collected from various habitats and geographic regions are necessary to explore how environmental selection and geographic isolation contribute to population differentiation.

Streptomycetes, well known for their prolific secondary metabolism, are filamentous bacteria with complex lifestyles (26, 27). Due to sporulation, *Streptomyces* strains are distributed worldwide and are often found in various ecological settings (28, 29). These traits make streptomycetes robust models for examining how geography and ecology influence population structure and evolution. In addition to their well-known edaphic and aquatic habitats, host-associated systems, notably insect-associated habitats, are also major habitats for *Streptomyces* (30). Stimulated by the discovery of new natural products, many previous studies investigated the defensive mutualism between *Streptomyces* and insects (31, 32). *Streptomyc*es can evolve to yield novel antibiotics in the niches of insect-*Streptomyces* symbioses (33, 34). Another study indicated that insect hosts feeding on plant biomass likely select symbiotic *Streptomyces* for increased cellulose-degrading activity (35). These findings suggest that interactions with insects might facilitate the diversification of *Streptomyces*. However, the evolutionary mechanisms underlying the transition between free-living and insect-associated lifestyles, especially how symbiotic interactions drive the population differentiation and speciation of streptomycetes, remain poorly understood.

We have been collecting conspecific *Streptomyces* strains all over the world for several years. Our previous studies of a collection of Streptomyces albidoflavus strains (with a genome-wide average nucleotide identity [ANI] of >98.15%) from diverse habitats have indicated that ecological adaptation plays an important role in shaping the population structure of this species (36, 37). Here, we studied another collection of 37 more divergent strains (with a genome-wide ANI of >96.93%), residing in both free-living (FL) and insect-associated (IA) habitats encompassing multiple spatial scales. This collection includes 35 conspecific isolates previously assigned to Streptomyces olivaceus (including the type strain *S. olivaceus* CGMCC 4.1369) based on multilocus sequence analysis and phenotypic characteristics (38) and two *S. olivaceus* isolates with ANI values of 98.59% and 98.60% to the type strain. We investigated the population genetic structure and gene flow pattern and whether the differentiation within *S. olivaceus* is driven by geographic isolation, ecological isolation, or perhaps both. We hypothesized that ecological factors play a dominant role in driving the genetic divergence of *S. olivaceus* and that ecological dispersal limitation exists between FL and IA habitat types. Furthermore, using comparative genomics and physiological characterization, we identified genes that may account for habitat adaptation and links between genomic and ecological variations.

## RESULTS

### *S. olivaceus* strains segregate into two habitat-associated and geographically disparate lineages.

**FIG 1:**
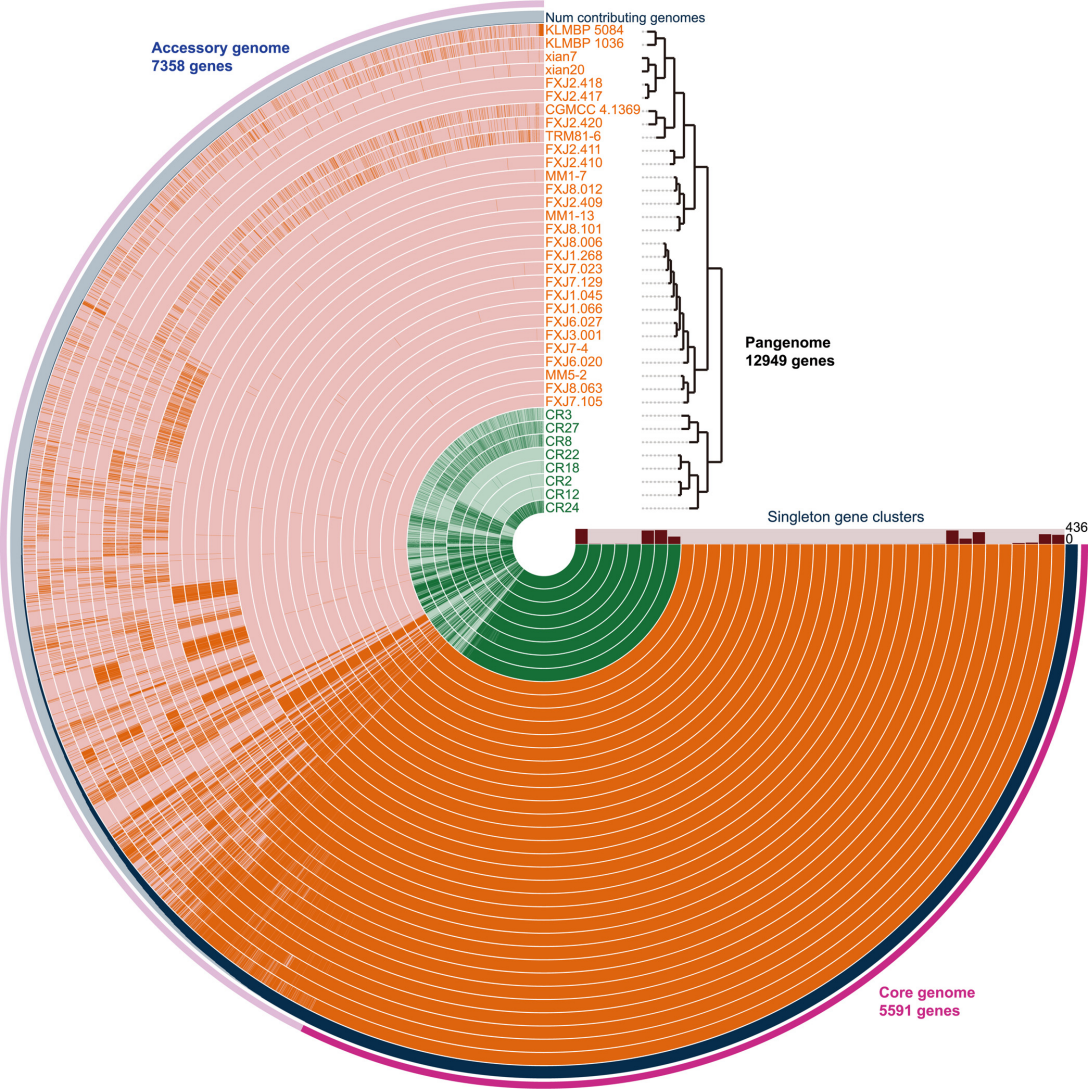
Pan-genome analysis of *S. olivaceus*. The tree on the top right displays the hierarchical clustering of 37 genomes based on the presence/absence patterns of 12,949 pan-genomic genes. The inner layers represent individual genomes with habitat sources colored green (insect-associated, IA) and orange (free-living, FL). In the layers, bars indicate the presence of genes in a given genome. In the outermost layer, the core (5,591 genes) and accessory (7,358 genes) genomes are indicated in dark and light pink, respectively. The second blue layer shows the number of genomes harboring each gene.

We collected 37 *S. olivaceus* strains that were isolated from diverse habitats and geographic locations (Table S1), comprising 29 strains from various soil and marine sources considered free-living (FL) habitats and 8 strains isolated from insect-associated (IA) habitats. The general genomic features of all strains are shown in Table S1. The genome size ranged from 8.11 to 8.66 Mb, and the number of coding sequences (CDSs) per genome was 6,966 to 7,512. Gene orthology analysis showed that the pan-genome consisted of 12,949 genes, including 2,973 strain-specific genes (Fig. 1). The core genome contained 5,591 genes (5,007 single-copy genes) and was predicted to encode 27 core secondary metabolite biosynthetic pathways (Table S2). The mean pairwise genome-wide ANI among the strains was 98.63%, with the lowest ANI of 96.93% observed between strains CR8 and KLBMP 5084 (Fig. S1A). The type strain *S. olivaceus* CGMCC 4.1369 shared an ANI of >97.16% with the other 36 strains. These ANI values were all above the typical prokaryotic species cutoff of 95 to 96% (4, 5). Furthermore, these strains formed a stable single-monophyletic cluster in the phylogenomic tree of the genus *Streptomyces* (Fig. S1B and C), sharing an ANI of <88.4% with other streptomycetes. These results support that the 37 strains belong to a cohesive unit, descending from a last common ancestor.

10.1128/mBio.02781-21.1 FIG S1 (A) Heatmap of genome-wide average nucleotide identities (ANI) among 37 *S. olivaceus* isolates. Strain numbers are colored by isolation sources: insect-associated (green) and free-living (orange). Cells in the heatmap represent pairwise ANI values with red color corresponding to an ANI of >95%, as shown in the color bar. Hierarchical clustering of ANI values in two dimensions is shown on the upper left of the heatmap. (B) Maximum likelihood phylogenetic tree generated from 138 single-copy actinobacterial marker genes of 238 *Streptomyces* type strains (after quality control of the genomes) and 37 *S. olivaceus* isolates. The scale bar represents nucleotide substitutions per site. Bootstrap values less than 70% are indicated by red dots at the nodes. The monophyletic clusters with more than 7 genomes are collapsed into lineage groups I to X. (C) Maximum likelihood phylogenetic tree generated from 138 single-copy actinobacterial marker genes of 996 *Streptomyces* strains (after quality control of the genomes) and 37 *S. olivaceus* isolates. Branch lengths are ignored to better illustrate the phylogenetic relationship. Bootstrap values less than 70% are indicated by red dots at the nodes. The monophyletic clusters with more than 9 genomes are collapsed into lineage groups A to O. Download FIG S1, TIF file, 2.2 MB. Copyright © 2022 Wang et al. 2022 Wang et al. https://creativecommons.org/licenses/by/4.0/ This content is distributed under the terms of the Creative Commons Attribution 4.0 International license.

10.1128/mBio.02781-21.6 TABLE S1 Genomic and geographic characteristics of *S. olivaceus* strains used in this study. Download Table S1, DOCX file, 0.05 MB. Copyright © 2022 Wang et al. 2022 Wang et al. https://creativecommons.org/licenses/by/4.0/ This content is distributed under the terms of the Creative Commons Attribution 4.0 International license.

10.1128/mBio.02781-21.7 TABLE S2 The core secondary metabolite biosynthetic gene clusters of *S. olivaceus* predicted using antiSMASH 5.0. Download Table S2, DOCX file, 0.03 MB. Copyright © 2022 Wang et al. 2022 Wang et al. https://creativecommons.org/licenses/by/4.0/ This content is distributed under the terms of the Creative Commons Attribution 4.0 International license.

**FIG 2:**
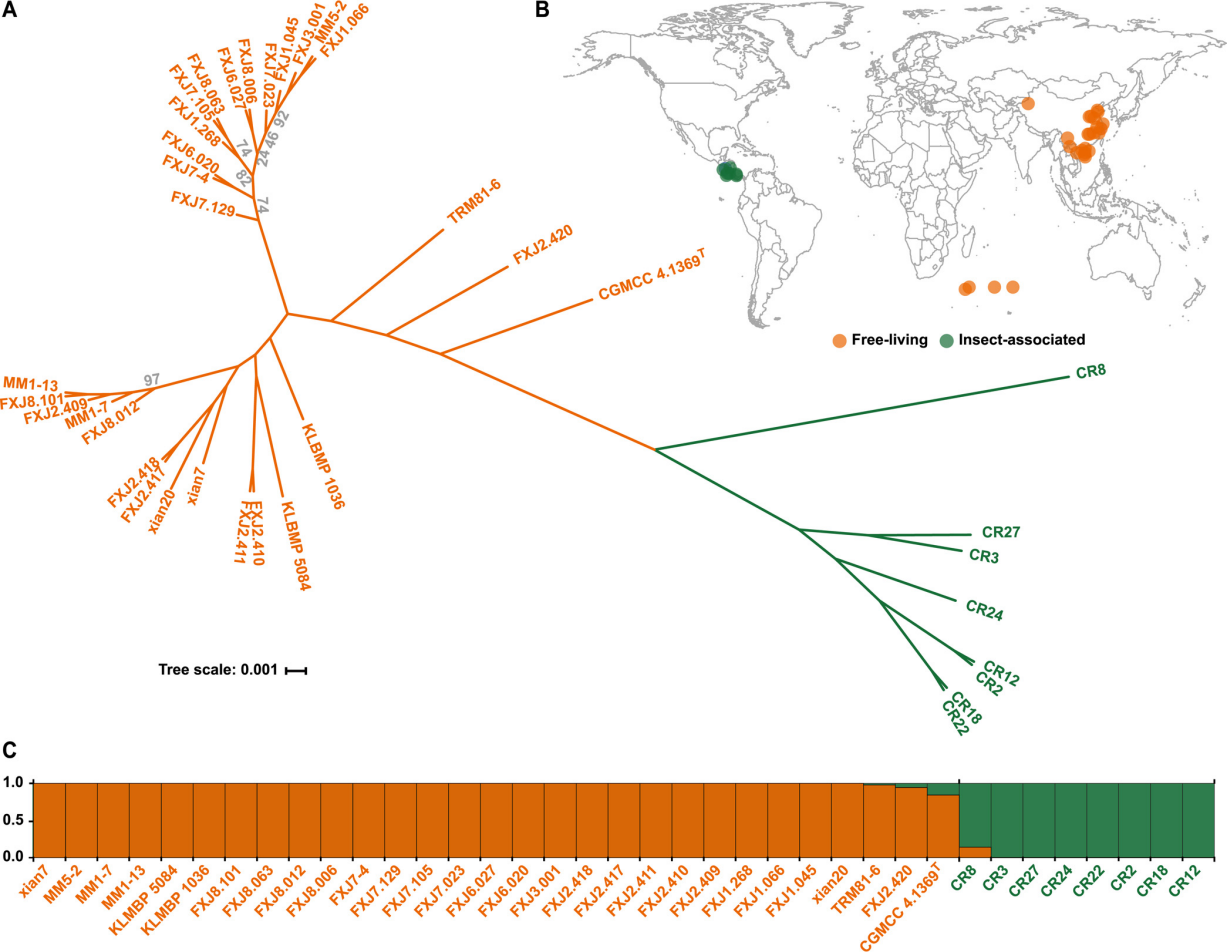
(A) Maximum likelihood phylogenetic tree generated from concatenated single-copy core genes of 37 *S. olivaceus* strains. The scale bar represents nucleotide substitutions per site. Bootstrap values less than 100% are shown in gray at the nodes. Clades are indicated by colors: clade FL (orange) and clade IA (green). The colors of strain numbers reflect their isolation sources: free-living (orange) and insect-associated (green). (B) The geographic distribution of isolates. Strain CGMCC 4.1369^T^ is not presented here because information on its geographic origin was unavailable. (C) Ancestral population structure of *S. olivaceus* reconstructed using fastSTRUCTURE. Bar plots show the proportion of each strain originating from two estimated ancestral populations.

Despite the high genetic relatedness of *S. olivaceus* strains, two robust clades were clearly identified by both hierarchical clustering analysis based on the pan-genome profile (Fig. 1) and phylogenetic analysis based on concatenated single-copy core genes (containing 4,948,371 nucleotides [nt]) (Fig. 2A). The two clades were clustered strictly according to habitat types (FL and IA), which overlapped with two roughly divided geographic regions, Costa Rica in the Western Hemisphere and China and the southern/southwest Indian Ocean in the Eastern Hemisphere (Fig. 2B). In particular, one clade consisted of 8 IA strains (clade IA) isolated from Costa Rica, sharing an intraclade ANI of 97.45 to 99.99%, and the other clade included the remaining 29 FL strains (clade FL) isolated from China and the southern/southwest Indian Ocean, sharing an intraclade ANI of 98.23 to 99.998%. In addition, fastSTRUCTURE (39) analysis inferred two ancestral populations (best K = 2, where K denotes the number of populations) consistent with the two clades (Fig. 2C). All these data indicate that *S. olivaceus* consists of two closely related but distinct sister lineages, an FL lineage and an IA lineage.

### Gene flow barriers between lineages.

To reveal the processes generating the differentiation between the FL and IA lineages, we investigated core-genome gene flow among *S. olivaceus* strains. First, we employed ClonalFrameML v1.12 (40) to quantify the degree of homologous recombination (Table 1). In the species and FL clade, recombination contributed only slightly more to the diversity than mutation (relative impact of recombination versus mutation on the per-site substitution rate [*r*/*m*] = 1.32 and 1.48, respectively). In contrast, we observed a strong recombination effect in clade IA (*r*/*m* = 3.95). Given that the impact of recombination might be underestimated among highly similar strains (41, 42), we repeated the analysis without the very closely related strains. As a result, the effect of recombination on diversity increased markedly in clade FL (*r*/*m* = 2.46) but remained relatively low in the species overall (*r*/*m* = 1.58), indicating higher recombination levels within than between the clades (Table 1). Furthermore, 1,252 (65.3%) and 853 (86.6%) recombination events in clades FL and IA, respectively, likely had within-clade and external (outside the clades) origins, representing the main sources of gene exchange in *S. olivaceus* (Fig. S2). Only 3.0% and 7.4% of recombination events in clades FL and IA, respectively, were explicitly predicted to have originated from the other clade. The evidently reduced interclade recombination events suggested restricted gene flow between the two clades.

**TABLE 1:** Recombination parameters of *S. olivaceus* quantified using ClonalFrameML v1.12

Clade	Events	*R/θ* ^*a*^	*δ* ^*b*^	*ν* ^*c*^	*r/m* ^*d*^
Clade IA (*n* = 8)	985	1.3693	88.1951	0.0327	3.9478
Clade FL (*n* = 29)	1,917	0.4951	83.9948	0.0357	1.4843
Clade FL subset^*e*^ (*n* = 11)	1,921	0.7650	96.1770	0.0334	2.4583
Total subset^*f*^ (*n* = 17)	2,761	0.5786	82.7363	0.0330	1.5817
Total (*n* = 37)	2,766	0.4752	75.8956	0.0365	1.3179

a Per-site rate of initiation of recombination relative to mutation.

b Mean length of DNA imported by homologous recombination.

c Divergence rate, per site, of DNA imported by homologous recombination.

d The relative impact of recombination versus mutation on the per-site substitution rate. Equal to (R/θ) × δ × ν.

e A set of clade FL strains without 18 FL strains that are collapsed into single nodes in Fig. 3A.

f A set of *S. olivaceus* strains without 18 FL strains and 2 IA strains that are collapsed into single nodes in Fig. 3A.

10.1128/mBio.02781-21.2 FIG S2 Core genome recombination in *S. olivaceus* detected using ClonalFrameML. Vectors indicate the sources and directions of the inferred recombinant regions. The numbers of recombination events and the percentages of the total recombination events in each clade are labeled at the vectors. “Internal (ambiguous)” indicates that recombinant fragments likely originated within clade FL or IA, but the source could not be explicitly assigned to a single clade. “External” indicates that recombinant fragments likely originated from outside clades FL and IA. Download FIG S2, TIF file, 1.2 MB. Copyright © 2022 Wang et al. 2022 Wang et al. https://creativecommons.org/licenses/by/4.0/ This content is distributed under the terms of the Creative Commons Attribution 4.0 International license.

To confirm the gene flow pattern, we employed PopCOGenT (6) to predict the contemporary recombination pattern and fine-scale population structure of *S. olivaceus*. This approach identified three separate populations, I to III (Fig. 3A). Population I corresponded well to clade FL and exclusively included most FL strains (except for two FL strains that were assigned as singletons). Interestingly, the IA strains were assigned to populations II and III, although they were isolated from a single insect species collected in the same area in Costa Rica. This result indicated limited recent gene flow between the FL and IA lineages and even within the IA lineage. The singleton nodes might imply underrepresented diversity due to limited sampling. In addition, as a complement to the overall and contemporary recombination analyses, we used fastGEAR to infer nonancestral (affected a subset of recipient lineages) and ancestral (shared by all strains in lineages) recombination (43). Notably, most recombination events were identified as nonancestral, including those between the clades; only a dozen events within clade IA were ancestral (Fig. 3B). Once again, the result that recombination was much more frequent within than between the clades confirmed the gene flow pattern (Fig. 3B).

**FIG 3:**
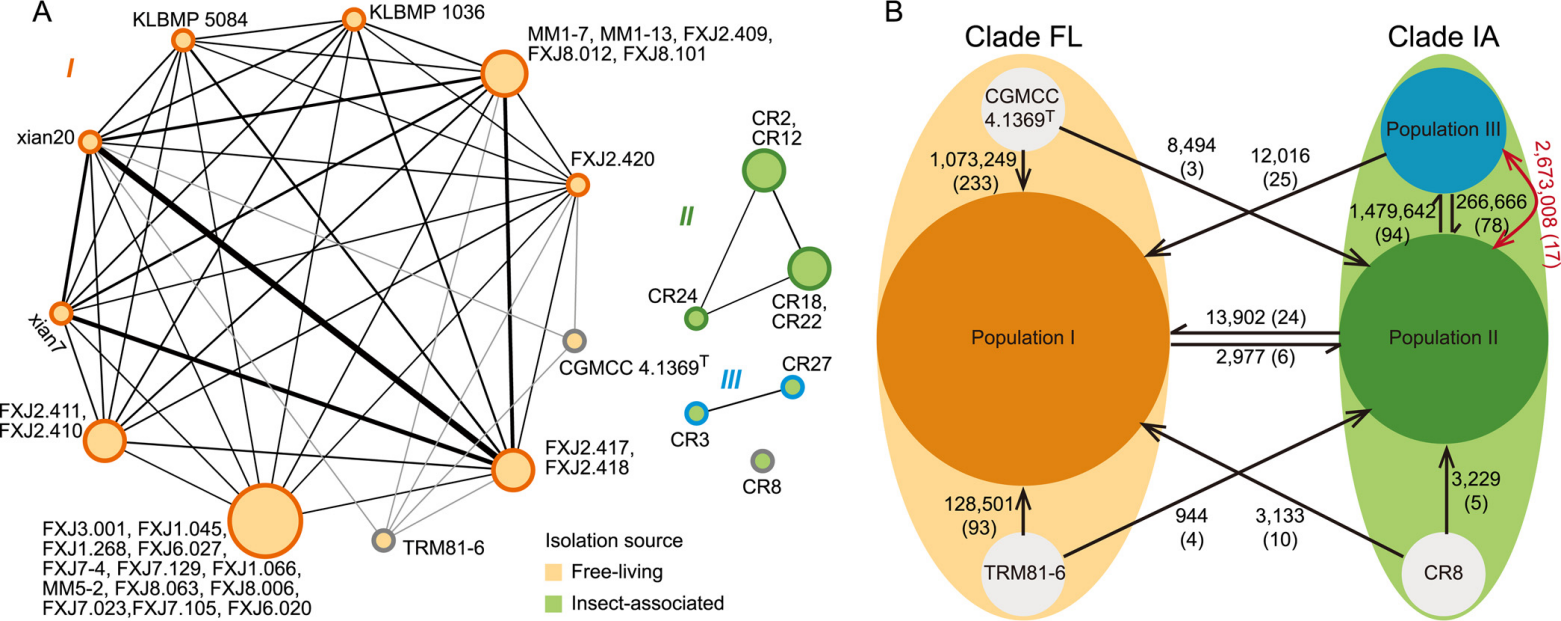
(A) Gene flow network and population structure identified with PopCOGenT. Edges represent the gene flow between genomes, with thicknesses corresponding to the amount of inferred gene flow and colors representing gene flow within (black) and between (gray) populations. Nodes are colored by isolation source, and node outlines are colored by population assignment: population I (orange), population II (green), population III (blue), and singletons (gray). Node size represents the number of clonal clusters (isolates that are too closely related are collapsed into a single node). (B) Diagram of recombination in the core genomes of *S. olivaceus* identified with fastGEAR. Population assignments were obtained from PopCOGenT. Black arrows indicate recent recombination events from donor to recipient populations. The red arrows indicate ancestral recombination events. The total length of recombinant regions (base pairs) and the numbers of recombination events are labeled on the vectors.

**FIG 4:**
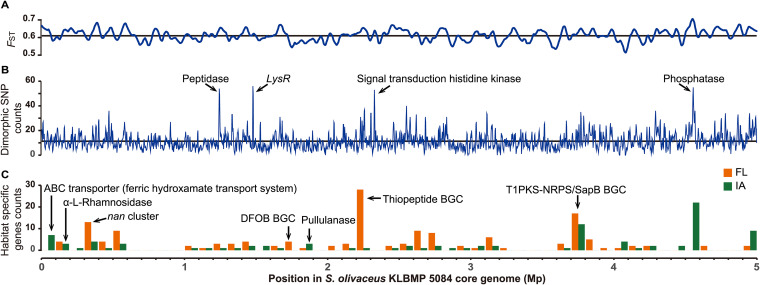
Genome differentiation of *S. olivaceus*. (A) Values of genetic differentiation (*F*_ST_) for 5-kb sliding windows moving in 2.5-kb steps are shown. This indicates genome-wide differentiation between clades FL and IA. The black line shows the mean *F*_ST_ value per 5 kb. (B) The numbers of clade-specific dimorphic SNPs within a 5-kb sliding window with a 2.5-kb overlap plotted along the core genome. The black line shows the average number of clade-specific SNPs per 5 kb. (C) Bar graph showing the distribution of clade-specific genes along the core genome. Each bar represents the number of genes within a 100-kb sliding window. BGC, biosynthetic gene cluster; DFOB, desferrioxamine B; *nan*, sialic acid catabolic gene cluster.

Moreover, we detected relatively high genome-wide interclade genetic differentiation (*F*_ST_) values (0.61 ± 0.06, mean ± standard deviation [SD]) (Fig. 4A), signifying strong genetic differentiation between the lineages. In addition, among 168,047 single nucleotide polymorphisms (SNPs), 11,994 were clade-specific SNPs with some alleles present in clade IA but others in clade FL. The clade-specific SNPs were distributed throughout the core genome, with only four small peaks (Table S3) containing four times the average SNPs (11.3 SNPs) in a 5-kb sliding window (Fig. 4B). This SNP distribution pattern provided additional evidence for genome differentiation and restricted gene flow between the lineages.

10.1128/mBio.02781-21.8 TABLE S3 Functional annotation of genes in the *S. olivaceus* genomic regions with four times the average clade-specific SNPs. Download Table S3, DOCX file, 0.03 MB. Copyright © 2022 Wang et al. 2022 Wang et al. https://creativecommons.org/licenses/by/4.0/ This content is distributed under the terms of the Creative Commons Attribution 4.0 International license.

### Geographic isolation versus ecological isolation.

Both ecological differentiation and limited gene flow caused by physical factors can generate population genetic patterns in bacteria (44). To examine the relative contributions of these processes in *S. olivaceus*, we considered both geographic and ecological information of the strains. FL strains were isolated from habitats spanning 0- to 107-m geographic distances, indicating an ambiguous biogeographical pattern. Additionally, the Mantel test for the 28 FL strains (without strain CGMCC 4.1681^T^ due to a lack of geographical location information) indicated no significant correlation between phylogenetic and geographic distances (*r *=* *0.072, *P = *0.621). These findings suggest that the movement of *S. olivaceus* is not limited among FL habitats on intercontinental scales. For example, soil isolates FXJ1.045 and FXJ1.066 were geographically separated from deep-sea isolate FXJ8.063 over 10^7^ m, while they were clustered into a single node in PopCOGenT analysis (Fig. 3A), indicating a recent dispersal event or rampant gene flow. Because geography was confounded with habitat type in our study, we performed partial Mantel tests for all strains, which revealed a significant effect of habitat type (*r *=* *0.935, *P < *0.001) but not geographic distance (*r* = −0.237, *P = *0.999) on phylogenetic distance.

Because the two clades reside in distinct ecological habitats, environmental selection may play an important role in genetic divergence, which could be proven by ecological specialization to different habitat types. To test this hypothesis, we performed principal-component analysis (PCA) on clusters of orthologous groups (COG) annotation profiles of the genomes to explore the functional differentiation in *S. olivaceus*. Two separate functional groups were identified, which displayed high congruency with the genetic structure (Fig. S3A), suggesting functional divergence between clades FL and IA. Moreover, the intraclade strains shared more accessory genes than the interclade strains (Fig. S3B), indicating that the accessory genome might carry functionally important genes that facilitate habitat adaptation and drive population differentiation.

10.1128/mBio.02781-21.3 FIG S3 (A) PCA analysis of COG profiles of *S. olivaceus* genomes. Each point represents a genome. Clades FL and IA are highlighted with ellipses in orange and green, respectively. (B) Heatmap of accessory genome similarities based on the 1-Jaccard distances (1-*d*). The tree on the left was derived from neighbor-joining analysis of the Jaccard distances. The isolation sources of strains are indicated in colors: insect-associated (green) and free-living (orange). (C) Numbers of clade-specific orthologous genes in different COG categories. Orange and green colors represent genes belonging to the free-living and insect-associated lineages, respectively. Download FIG S3, TIF file, 2.5 MB. Copyright © 2022 Wang et al. 2022 Wang et al. https://creativecommons.org/licenses/by/4.0/ This content is distributed under the terms of the Creative Commons Attribution 4.0 International license.

### Functional divergence in the accessory genome between lineages.

To further investigate functional differentiation in the accessory genomes, we explored the clade-specific genes that are universal in one clade but absent in the other, which might contain candidate habitat-adaptive genes. We found 150 and 98 orthologous genes (OGs) that were specific to clades FL and IA, respectively (Table S4), and distributed along the genome (Fig. 4C). Based on COG annotation, 175 clade-specific OGs (70.6%) were functionally categorized (Fig. S3C). Among these, 42 OGs, the highest number of OGs, were assigned to the COG class transcription (COG K), suggesting that transcriptional regulation facilitates *S. olivaceus* survival in specific habitats.

10.1128/mBio.02781-21.9 TABLE S4 Functional annotation of clade-specific genes in *S. olivaceus*. Download Table S4, DOCX file, 0.06 MB. Copyright © 2022 Wang et al. 2022 Wang et al. https://creativecommons.org/licenses/by/4.0/ This content is distributed under the terms of the Creative Commons Attribution 4.0 International license.

**FIG 5:**
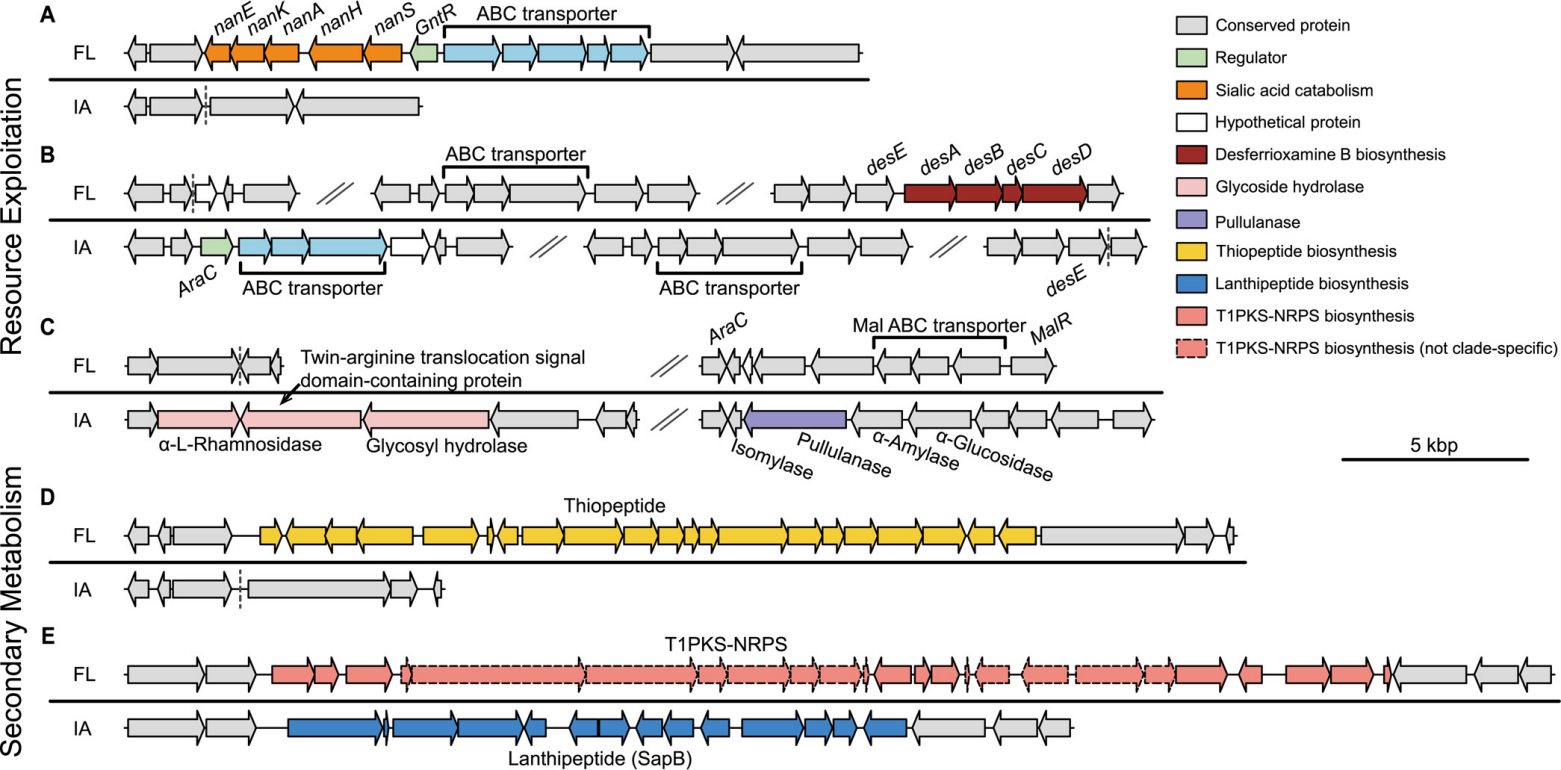
Genetic organizations of the regions containing clade-specific genes involved in resource exploitation and secondary metabolism. Conserved genomic regions flanking these genes are colored in gray. (A) The *nan* gene cluster for sialic acid catabolism. (B) The transport system for the hydroxamate-type iron complex and the biosynthesis gene cluster of desferrioxamine B. (C) The region containing a gene annotated as α-l-rhamnosidase and the region containing a putative pullulanase gene in a starch catabolic gene cluster. (D) The biosynthesis gene cluster of a thiopeptide. (E) The biosynthesis gene clusters of a T1PKS-NRPS and SapB.

More detailed analyses of the functions of the clade-specific OGs revealed that many of them were involved in resource exploitation and secondary metabolism. These OGs were often physically adjacent and clustered into genomic regions. Particularly interesting was the distribution of the sialic acid catabolic gene cluster (*nan*) (from KLBMP 5084_605 to KLBMP 5084_615) (Fig. 5A), which was reported as an FL habitat-associated genomic region in our previous study of *S. albidoflavus* (37). Two other interesting regions were involved in the uptake of iron, encoding an ABC transporter used for hydroxamate-type iron complex import (from CR12_85 to CR12_88) and a biosynthetic pathway for desferrioxamine B (DFOB) (from KLBMP 5084_2430 to KLBMP 5084_2433) (Fig. 5B). Although DFOB is a hydroxamate-type siderophore widespread in *Streptomyces* (45), all IA strains retained the complete transport system of DFOB but had lost its biosynthetic genes (46). In addition to the transport system that has high homology with its counterpart in FL strains, IA strains also had another hydroxamate transport system that was clade-specific (from CR12_85 to CR12_88). Besides these gene clusters, several clade-specific genes may also confer benefits to the populations that harbor them. For example, two genes (CR12_143 and CR12_1679) specific to IA strains were annotated as α-l-rhamnosidase and pullulanase, respectively (Fig. 5C), which might help break down complex carbohydrates such as naringin, glycogen, and amylopectin (47, 48). Further analysis of the genomic regions flanking these genes showed that the pullulanase gene was in a starch catabolic gene cluster that was conserved across all strains (Fig. 5C), indicating a gene loss event in FL strains.

Moreover, there were two clade-specific regions harboring secondary metabolite biosynthetic gene clusters (BGCs). One region was specific to FL strains (from KLBMP 5084_3249 to KLBMP 5084_3269) and predicted to encode a thiopeptide product (Fig. 5D), which shared little homology with known BGCs in MIBiG (49). The other was a versatile region with four genotypes (Fig. 5E). One genotype (from CR12_5271 to CR12_5284) was specific to IA strains, encoding a BGC displaying high homology with SapB (78%), a lanthipeptide that functions as a biological surfactant and facilitates the formation of aerial mycelium (50). The other three genotypes existed in FL strains, including several FL-specific genes (from KLBMP 5084_5460 to KLBMP 5084_5462, from KLBMP 5084_5471 to KLBMP 5084_5473, and from KLBMP 5084_5479 to KLBMP 5084_5483). These genes were part of a T1PKS-NRPS-type BGC, which had no significant hits in MIBiG. Overall, the clade-specific genes tend to be involved in habitat adaptation, indicating different evolutionary trajectories between the FL and IA lineages.

### Evidence for habitat-associated physiological differentiation.

**FIG 6:**
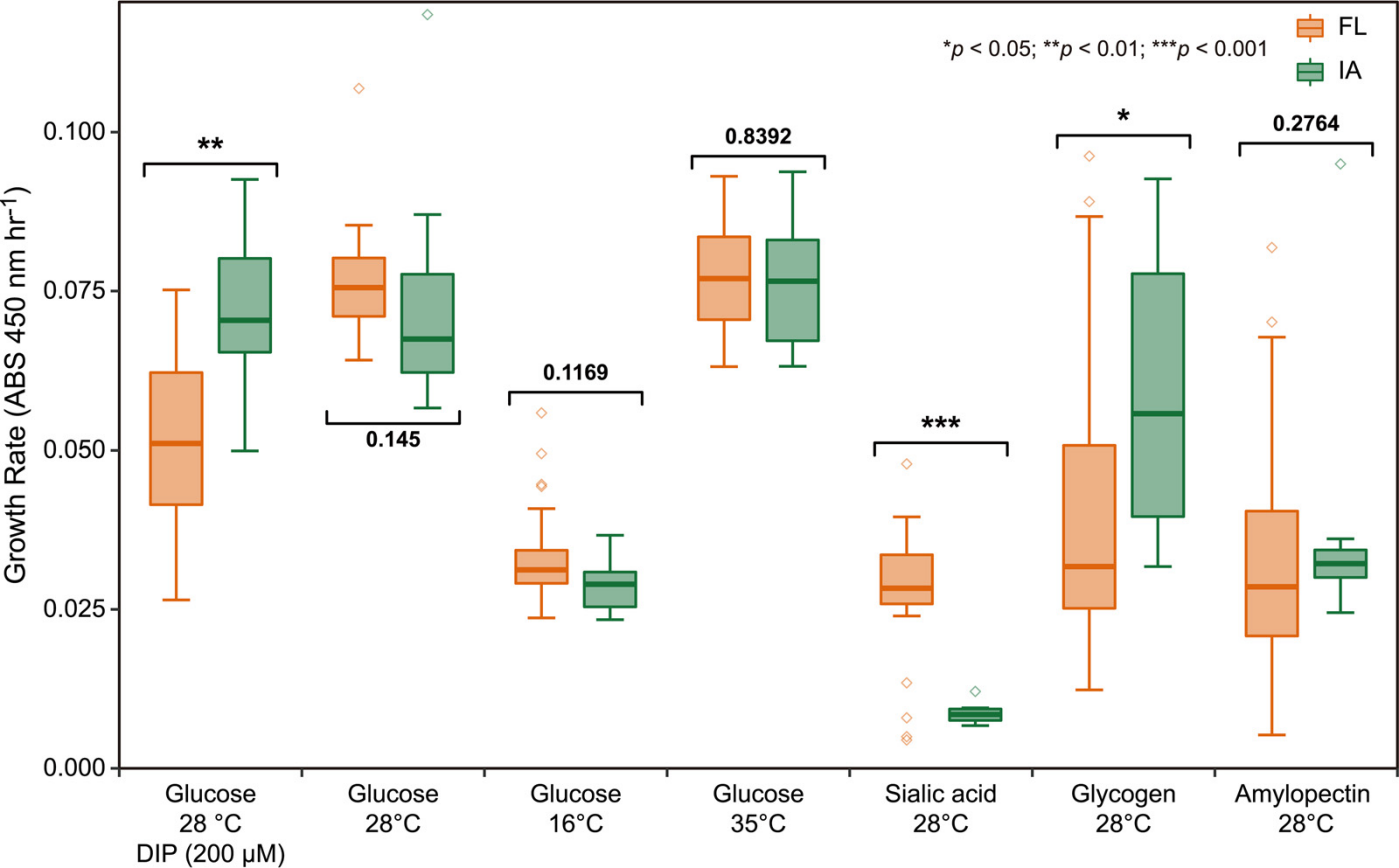
Growth rates (ABS 450 nm h^−1^) of the two lineages under different iron availability, temperature, and sole carbon source conditions, in which three replicates were performed for each strain. The free-iron-limited condition was simulated by adding the iron chelator DIP. The box shows the upper and lower quartiles, and the short line within the box represents the median. The error bar depicts the 1.5 interquartile range above the upper box boundary and below the lower box boundary. Outliers are plotted as individual points. *P* values were determined using a Mann-Whitney *U* test.

Given that little free iron is available in host environments (51), the ability to scavenge this element from free-iron-deficient environments is essential for the survival of host-associated microorganisms. We performed an absorbance-based assay to quantify the growth rates of *S. olivaceus* strains under different iron availability conditions. Low iron availability was simulated by adding the iron chelator 2,2′-bipyridyl (DIP) to the medium. FL and IA strains had nearly equal growth rates on the control medium without DIP, whereas IA strains grew significantly faster than FL strains on the medium with DIP (Mann-Whitney *U* test, *P = *0.002) (Fig. 6, Table S5). This result indicates that IA strains have greater average fitness than FL strains under free-iron-limiting conditions, which might be the result of adaptation to insect-associated habitats.

10.1128/mBio.02781-21.10 TABLE S5 Growth rates (ABS 450 nm h^−1^) for *S. olivaceus* under different iron availability, temperature, and sole carbon source conditions. Download Table S5, DOCX file, 0.03 MB. Copyright © 2022 Wang et al. 2022 Wang et al. https://creativecommons.org/licenses/by/4.0/ This content is distributed under the terms of the Creative Commons Attribution 4.0 International license.

The growth of *S. olivaceus* under different temperature conditions was tested using the same approach. We found no statistically significant difference in growth rates between clades FL and IA at 16, 28, or 35°C (Fig. 6, Table S5). Considering that the strains were isolated from different latitudes, we separated them into tropical (16 strains) and subtropical (20 strains) groups. However, the two groups showed no significant differences in growth rates at different temperatures (Fig. S4). Motivated by the prediction of clade-specific genes involved in carbohydrate utilization, we compared the growth rates of FL and IA strains on media containing different sole carbon sources. As expected, sialic acid and glycogen differentiated the two clades very well (Fig. 6, Table S5). Sialic acid could be utilized only by FL strains (Mann-Whitney *U* test, *P < *0.001), while glycogen supported faster growth of IA strains than of FL strains (Mann-Whitney *U* test, *P = *0.037) (Fig. 6, Table S5). However, amylopectin did not differentiate the two clades (Mann-Whitney *U* test, *P = *0.276). Neither carbohydrate utilization nor iron acquisition capacity differentiated the tropical and subtropical groups (Fig. S4).

10.1128/mBio.02781-21.4 FIG S4 Growth rates (ABS 450 nm h^−1^) of tropical and subtropical groups under different iron availability, temperature, and sole carbon source conditions, in which three replicates were performed for each strain. The free-iron-limited condition was simulated by adding the iron chelator 2,2′-bipyridyl (DIP). The box shows the upper and lower quartiles, and the short line within the box represents the median. The error bar depicts the 1.5 interquartile range above the upper box boundary and below the lower box boundary. Outliers are plotted as individual points. *P* values were determined using a Mann-Whitney *U* test. Download FIG S4, TIF file, 2.7 MB. Copyright © 2022 Wang et al. 2022 Wang et al. https://creativecommons.org/licenses/by/4.0/ This content is distributed under the terms of the Creative Commons Attribution 4.0 International license.

## DISCUSSION

Different evolutionary forces interact in nature. Here, we aimed to identify the processes responsible for the differentiation of two closely related but genetically distinct *Streptomyces* lineages (FL and IA), considering both geographic and ecological factors. One possible explanation for this genetic pattern is geographic isolation (12), which has been observed in the hyperthermophilic archaeon *Sulfolobus* (9), the bacterial pathogen Salmonella (52), and the free-living soil bacterium *Curtobacterium* (11). This scenario, however, did not explain the dispersal limitation observed here. The long-distance dispersal and gene flow of *S. olivaceus* were not limited at intercontinental scales (over 10^7^ m). Notably, although the two clades were isolated at different latitudes, growth under different temperatures did not differentiate them.

A promising interpretation for the differentiation of the two lineages is ecological divergence, which has been proven to be a driver of bacterial speciation (11, 25, 53). In contrast to previous studies of ecological speciation, ours did not reveal obvious genomic “islands” or even “continents” in the core genomes, which is a signature of ecological differentiation (19, 44, 53). This is because these genomic patterns persist for only a short time after the population splits (19, 54). The two lineages of *S. olivaceus* diverged approximately 6.91 million years (my) ago (confidence interval, 4.74 to 10.09 my ago; Fig. S5), which is comparable to the time span (8 ± 2.5 my) required for *Streptomyces* species to accumulate 1% core gene amino acid divergence (55). Combined with the relatively high number of clade-specific dimorphic SNPs and the less evident dimorphic SNP peaks in *S. olivaceus* genomes (Fig. 4B), these results suggest that after a long period of ecological isolation and dispersal limitation, the initial recombination barriers have spread throughout the genomes, resulting in two young sister species.

10.1128/mBio.02781-21.5 FIG S5 Molecular clock for the genus *Streptomyces*. The phylogeny was generated using 94 universally conserved housekeeping proteins in the “core bacterial protein” set (GenProp0799). The two major monophyletic clades of *Streptomyces* are marked clade I and clade II as previously reported (55). The free-living and insect-associated lineages of *S. olivaceus* are in orange and green fonts, respectively, in clade II. Branch lengths indicate RelTime-estimated divergence times. Bootstrap values less than 100% are shown in gray at the nodes. Download FIG S5, TIF file, 1.4 MB. Copyright © 2022 Wang et al. 2022 Wang et al. https://creativecommons.org/licenses/by/4.0/ This content is distributed under the terms of the Creative Commons Attribution 4.0 International license.

As massive gene flux may occur within natural *Streptomyces* populations (56), the speciation of *S. olivaceus* was likely initiated by gene gain or loss in the ancestral population, which allowed the mutants to colonize a novel habitat (53). The observation that most of the clade-specific regions were flanked by conserved core genes, combined with the absence of mobile genetic elements around these regions (Fig. 5), suggests an important role of homologous recombination in the gene gain or loss. Subsequent homologous recombination, possibly combined with vertical inheritance, might have facilitated the sweep of habitat-specific genes among new populations, leading to dispersal limitation across habitats due to fitness trade-offs. Thus, the reduced encounters and accumulated sequence divergence between populations could gradually enhance ecological barriers to gene flow and eventually result in genetic isolation across the genome. The identification of habitat-specific genes in accessory genomes and the observation of fitness trade-offs under different environmental conditions provide strong evidence for this explanation. Although we could not determine which genes initiated ecological speciation, our analysis identified a set of habitat-adaptive genes.

Most noteworthy is the *nan* gene cluster for sialic acid catabolism. Sialic acids, a family of nine carbon monosaccharides, are basic components of some cell surface glycoproteins and glycolipids (57). Sialic acids play pivotal roles in the development and function of the nervous system in insects (58–60). Therefore, sialic acid catabolism seems to be harmful to insect hosts, which is in line with the distribution pattern of the *nan* cluster and corresponding functional corroboration in *S. olivaceus* (Fig. 5 and 6). Strains without the *nan* cluster could easily colonize and adapt to insect-associated niches, while strains with the ability to utilize sialic acids may perform better within the complex and varying free-living environment. As a consequence, the dispersal from FL to IA habitats might be limited, and vice versa. These results confirmed our previous identification of the *nan* cluster as a habitat-adaptive region in *Streptomyces* species (37), providing compelling evidence of convergent evolution toward this successful ecological strategy (61, 62). Most likely, as compensation for the inability of IA strains to utilize sialic acids, they possess pullulanase genes and utilize glycogen (not amylopectin), the major carbohydrate reserve in insects (63), more efficiently than FL strains (Fig. 6). The preference for different carbon sources illustrates habitat partitioning between the two lineages.

The different growth rates of the two lineages under free-iron-limited conditions were also linked to fitness differences, providing additional evidence for ecological adaptation. Competition for iron resources is an important aspect of microbial interactions (64, 65), especially within host environments, where free iron is generally deficient (51). Hence, the loss of DFOB biosynthetic genes but retention of the DFOB transport system and the acquisition of another transporter for the hydroxamate-type iron complex might enable IA strains to pirate siderophores for growth (16, 66, 67). This is an energy-efficient strategy consistent with the Black Queen Hypothesis (68). Based on the fact that DFOB is a common hydroxamate-type siderophore and can be accessed from the surrounding community, this strategy confers IA strains selective advantages in environments with fierce iron competition. Furthermore, we identified four core siderophore BGCs in *S. olivaceus* genomes, two of which are hydroxamate-type siderophores (aerobactin-like and coelichelin) (Table S2). How these BGCs are regulated is unclear, but the observation that IA strains have significantly higher growth rates than FL strains under iron-limiting conditions indicates that the former may produce more siderophores and transporters and consequently have greater fitness than the latter in free-iron-deficient environments. Together, the results suggest that the different iron acquisition abilities between the two clades also contribute to ecological divergence and dispersal limitation.

In addition to adaptation to insect-associated environments, IA strains, in turn, might protect their insect hosts by producing detoxifying enzymes. For example, the clade IA-specific gene CR12_143 encodes putative α-l-rhamnosidase, an enzyme that cleaves terminal α-l-rhamnose from a large number of natural glycosides, e.g., naringin, rutin, and diosmin. These flavonoids mediate the defense response in plants, and the removal of rhamnose from their skeleton would suppress this process (47). Thus, the IA strains might provide their insect hosts counterdefenses against plant toxins. Moreover, secondary metabolites, which can serve as weapons and signal molecules in microbial interactions (69), may provide producers selective advantages. For instance, the FL-specific BGCs thiopeptide and T1PKS-NRPS might contribute to the survival of FL strains by mediating local ecological interactions in the complex community.

Although the genome-wide ANI (96.93 to 99.998%) of *S. olivaceus* meets the standard threshold (95 to 96%) for prokaryotic species delineation (4, 5), the population structure, gene flow pattern, and ecological distribution and divergence results strongly indicate that the two clades represent independent lineages. These results further lead to an important question about whether the two lineages have diverged to the extent that they can represent two distinct species. When a species designation is questioned, multiple approaches should be taken into consideration. According to the unified species concept, two newly divergent lineages could be considered two species if they are evolving separately (70). In our case, despite the markedly restricted recent gene flow, low-level nonancestral recombination was still detected between the two lineages (Fig. 3), suggesting that they probably represent two incompletely separated species (71). Moreover, the two lineages are indistinguishable in the vast majority of phenotypic properties (38; unpublished data) and are far more distant from other streptomycetes than from each other in the genome-based *Streptomyces* phylogeny (Fig. S1B and C). All these findings, combined with the genetic and ecological distinction of the two lineages, support that they are recently diverged species that may still be in the gray zone of speciation described by de Queiroz (70). Although a universal ANI cutoff is taken as part of the criterion for segregating prokaryotic taxa, this approach is not suitable for the most recent products of speciation (72). Consequently, we propose to use the Open Nomenclature qualifier “species complex” (73) to accommodate the *S. olivaceus* strains. The FL lineage turns out to represent *S. olivaceus sensu stricto*, while the IA lineage may represent a cryptic species (74, 75) that needs further taxonomic investigation for a formal description.

Consistent with our previous findings in *S. albidoflavus*, the population structure of the *S. olivaceus* species complex was also associated with the change between FL and IA lifestyles. Although *S. albidoflavus* and *S. olivaceus* have been separated for more than 100 my (55), their similar evolutionary patterns highlight the potential role of lifestyle transitions in the divergence of streptomycetes. This is strengthened by the facts that *S. albidoflavus* is still at an advanced stage of speciation (37), while *S. olivaceus* has undergone recent speciation. Ecological interactions between microorganisms and insects, including nutritional and defensive interactions, have been described extensively (76, 77). The best-studied example is the insect-fungus agricultural symbiosis, which has undergone coevolution over tens of million of years (78). The domestication of fungi has involved transitions from loose cultivation to irreversible domestication (78), which is combined with the loss of some carbohydrate-degrading enzyme genes and restricted gene flow between fungal cultivars and their FL relatives (79). Similar evolutionary trajectories were found in our studies (i.e., gene loss and reduced gene flow). Intriguingly, the convergent strategy of sialic acid catabolism loss in both the *S. albidoflavus* and *S. olivaceus* clades associated with insects might indicate coevolution between *Streptomyces* species and their insect hosts. In addition to this gene cluster, a series of habitat-adaptive genes, such as genes involved in glycogen and iron utilization, collectively contributed to the transition between FL and IA lifestyles. Taken together, our results indicate that interactions with insects facilitate diversification and ecological divergence in *Streptomyces*. Hence, there is a great need to consider insect-associated habitats, one of the major sources of *Streptomyces*, when studying *Streptomyces* speciation.

Our study has some limitations. First, clade IA included a relatively small number of isolates, and the diversity of this clade might be underestimated, as observed in the inference of recombination origins. More isolates from IA sources should be included in future studies. Another potential limitation is that clades FL and IA are not sympatric. Although we reasoned that free-living *S. olivaceus* is not geographically restricted at intercontinental scales, there is no direct evidence to exclude the effect of geographic isolation. Adding sympatric but ecologically diverged conspecific isolates to the study will be required to clarify this question, although that previous isolation did not yield *S. olivaceus* strains from Chinese insects or Costa Rican soils. Other abiotic factors and biotic variables such as interactions with local communities might also impose selection pressure on the strains. Nevertheless, our findings highlight the roles of habitat adaptation and divergence in the origin and maintenance of population structure in *Streptomyces*. Further studies focusing on closely related isolates with detailed environmental metadata are likely to provide a better understanding of ecological adaptation and its contribution to the speciation and diversity of bacteria.

## MATERIALS AND METHODS

### Isolate collection.

A total of 37 *S. olivaceus* strains were used in this study, which were isolated from diverse habitats at multiple spatial scales (Table S1) and categorized into two habitat-associated groups, FL and IA. The FL group encompassed 19 strains isolated from soil and soil-associated habitats in China and 10 marine isolates derived from Chinese seas and the Indian Ocean. The IA group comprised 8 strains isolated from imperial moths (Eacles imperialis, Saturniidae) collected in Costa Rica.

### Genome sequencing, assembly, and annotation.

One complete reference genome (KLBMP 5084) was downloaded from the National Center for Biotechnology Information (NCBI; https://www.ncbi.nlm.nih.gov/) database. For the remaining 36 strains, short-insert paired-end libraries were constructed and sequenced by Novogene Bioinformatics Technology Co., Ltd. (Beijing, China), using the Illumina HiSeq 2500 system. All good-quality paired reads were assembled using SOAPdenovo (80). All genome accession numbers are listed in Table S1.

All genomes were processed using Prodigal v2.6.2 with default parameters to predict coding sequences (CDSs). The CDSs were annotated with the COG 2003–2014 (81), Pfam 31.0 (82), and NCBI nonredundant (NR; accession date, 10 April 2019) (83) databases. Secondary metabolite biosynthetic gene clusters (BGCs) in each genome were predicted via antiSMASH v5.0 (84). The pairwise genome-wide ANI was calculated using pyANI (85).

### Pan-genome analysis and phylogenetic inference.

Orthologous groups (OGs) were identified using OrthoMCL algorithms (86) implemented in the Anvi’o pan-genomic workflow v6.2 (87) with BLAST 2.7.1+ (88), and the pan-genome results were visualized using “anvi-display-pan.” Single-copy core genes, which were defined as OGs with exactly one copy in each genome, were extracted from the “anvi-summarize” results. Each single-copy core OG was aligned with MUSCLE v3.8.1551 (89) at the amino acid level and then converted to nucleotide alignment through PAL2NAL v14 (90). Poorly aligned regions were trimmed using Gblocks v0.91b (91) with –t = c. The resulting alignments were concatenated with a custom Python script to obtain core genome sequences. A phylogenetic tree based on the single-copy core genomes was reconstructed using FastTree v2.1.10 (92) under the general time reversal model and visualized using iTOL (93). The population structure was predicted from the single nucleotide polymorphism (SNP) data set of single-copy core genomes using fastSTRUCTURE (39) with runs for population numbers (K values) of 2 to 10. The approximate divergence times for two clades was estimated using RelTime (94) as previously described (55). In addition, the phylogenomic tree of the genus *Streptomyces* was constructed to examine the phylogenetic status of the 37 strains using the pipelines described by Li et al. (95). Briefly, all genomes assigned to *Streptomyces* by the EzBioCloud database (96) (accession date, 24 September 2021) were downloaded from the NCBI genome FTP site (https://www.ncbi.nlm.nih.gov/genomes). After quality control, 996 genomes, together with the 37 *S. olivaceus* genomes, were subjected to the GToTree (97) workflow to identify the set of 138 actinobacterial marker genes, which was then used to generate the *Streptomyces* phylogeny.

### Recombination and gene flow analyses.

ClonalFrameML v1.12 (40) was used to detect and quantify homologous recombination. Specifically, recombination events among all 37 isolates and among the phylogenetic clades were reconstructed using core genome alignments. For each recombination event, genetic distances between the recombinant fragments and their homologous sequences from all nodes and leaves were calculated with the R package APE (98) to infer the likely origin of recombination, as described in a previous study (99). The clustering algorithm PopCOGenT (6) was used to detect recent gene flow and the contemporary boundaries of gene flow units that were defined as ecologically relevant populations. FastGEAR (43) was used to identify nonancestral and ancestral recombination events, using the core genome alignments of all 37 isolates and the population assignment result of PopCOGenT as input. Additionally, *F_ST_* values of the core genome in 5-kb sliding windows (2.5-kb overlap) were calculated using the R package PopGenome (100).

### The contribution of geography and ecology to population differentiation.

The pairwise geographic distances (if available) and phylogenetic distances based on the core genome between isolates were calculated using the R packages geosphere (101) and APE, respectively. In the matrix of habitat differences, all within-habitat-type pairwise comparisons were assigned a value of 0, whereas all FL versus IA pairwise comparisons were assigned a value of 1. The effects of geography and ecology on population structure were examined using Mantel and partial Mantel tests in the R package VEGAN (102) with 10,000 permutations. Mantel tests were performed between pairwise phylogenetic distances and geographic distances for the FL group only. Partial Mantel tests were performed for the whole species to test the effect of one factor while controlling for the other factor. This analysis was necessary given that the habitat types and sampling locations were confounded in our study.

### Identification and analysis of ecologically relevant variants.

Principal-component analysis (PCA) of COG annotation profiles of *S. olivaceus* genomes was performed using the prcomp function in R. The Bray-Curtis distance matrix, used as an input file for PCA, was calculated from COG annotation profiles after Hellinger transformation with the R package VEGAN. To estimate the similarity of flexible genomes, the Jaccard distances (*d*) based on the dispensable gene 0/1 matrix were calculated with vegdist in the R package VEGAN. These distances were then used as input to PHYLIP v3.698 (http://evolution.gs.washington.edu/phylip/) to generate a neighbor-joining tree. A heatmap of Jaccard similarity (1-*d*) was visualized with the R package pheatmap (https://github.com/raivokolde/pheatmap). Habitat-specific accessory genes and SNPs were identified using a custom Python script, with the pan-genome presence/absence matrix and the SNP file extracted from core genomes used as inputs, respectively.

### Evaluation of growth under different conditions.

To evaluate the growth of strains under different conditions, an absorbance-based approach described in a previous study (23) was used with a few modifications. In brief, a 5-μL spore suspension (10^5^ CFU/mL) of each strain was plated as a lawn onto 150 μL solid medium within 96-well plates, and the absorbance at 450 nm was measured every half an hour with a CLARIOstar Plus plate reader (BMG Labtech, Ortenberg, Germany) for 3 to 5 days. The change in absorbance was plotted against time to create a growth curve, and the growth rate was determined as the slope of the curve in the linear range. To test for differences in carbon source utilization among strains, we used basal mineral salt agar medium (MM) (103) supplemented with a final concentration of 1% sole carbon sources, including *N*-acetylneuraminic acid, glycogen, and amylopectin. Growth on MM was used as a negative control, whereas growth on MM supplemented with glucose was used as a positive control. To simulate free-iron-limited conditions, the iron chelator 2,2′-bipyridyl (DIP) was added to MM agar containing 1% glucose to a final concentration of 200 μM. Control experiments were carried out using the same medium without the addition of DIP. In addition, the growth rates at different temperatures (16 to 35°C) were measured using MM agar containing 1% glucose. All growth assays were performed in triplicate.

### Data availability.

The genome accession numbers for the strains in this study are listed in Table S1.
