# Supplementary figures and images for: Habitat Adaptation Drives Speciation of a Streptomyces Species with Distinct Habitats and Disparate Geographic Origins
Source: mBio. 2022 Jan 11;13(1):e02781-21. doi: 10.1128/mbio.02781-21 (PMC8749437; doi:10.1128/mbio.02781-21)

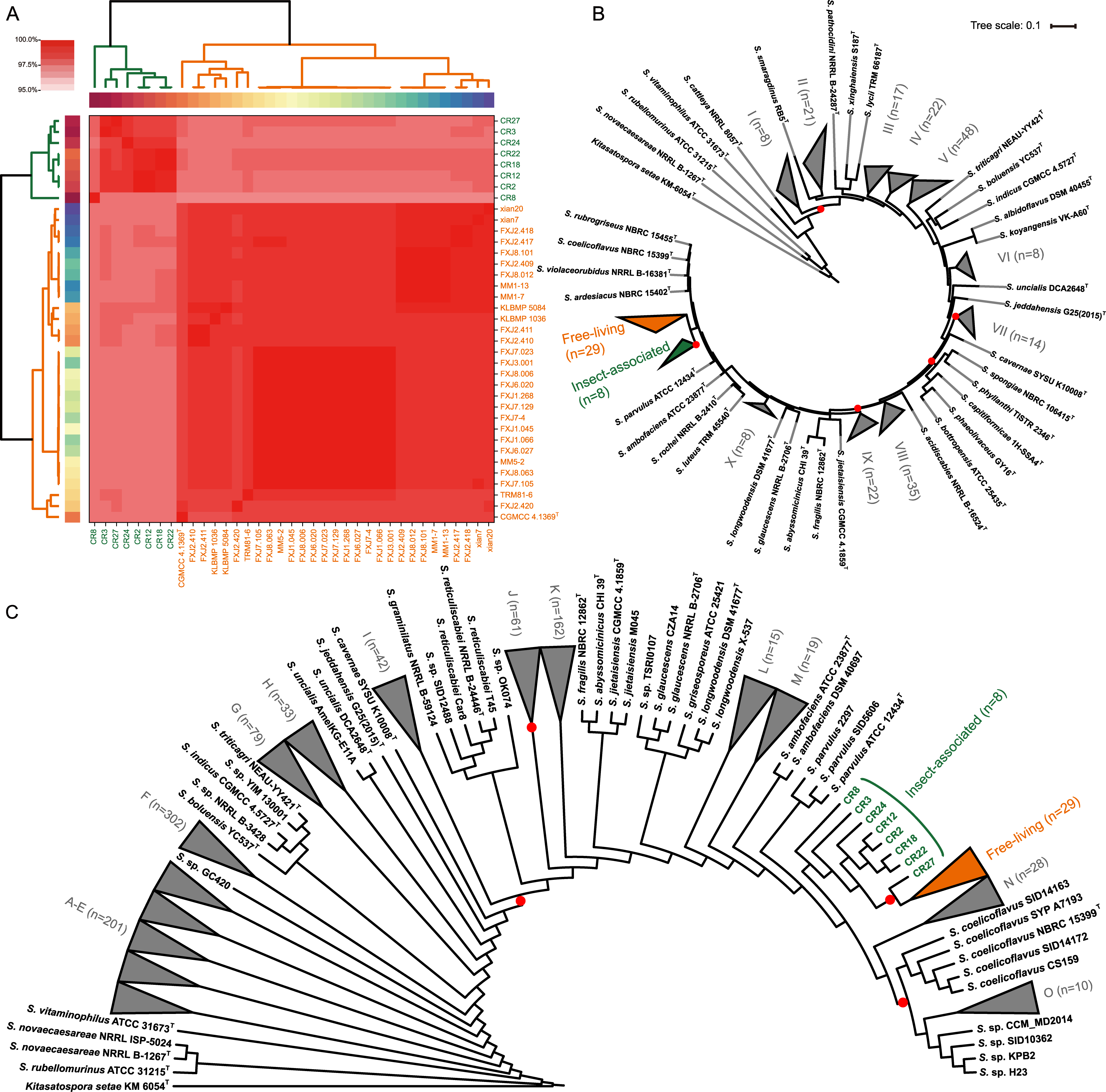

Supplement: FIG S1 [file mbio.02781-21-sf001.tif]

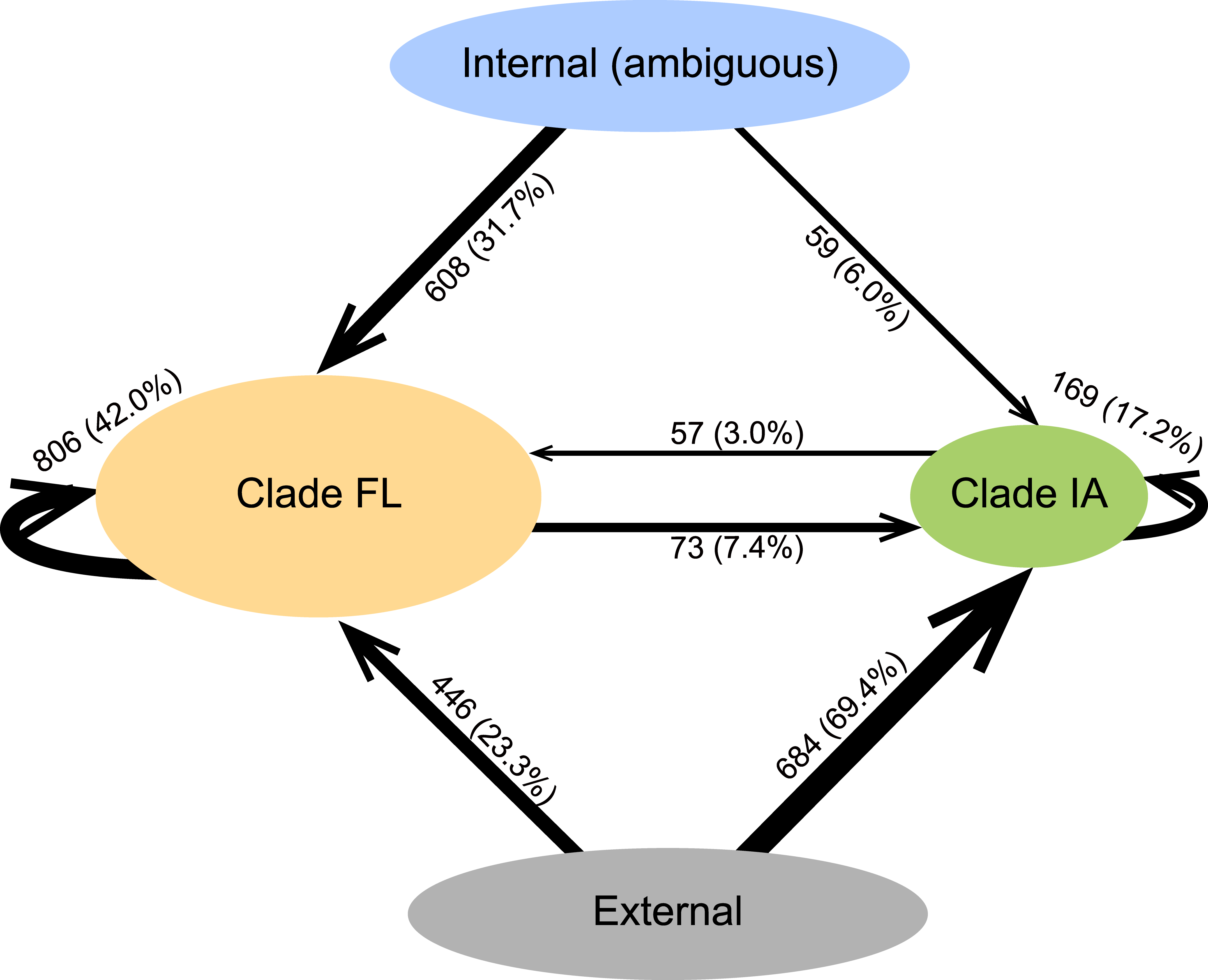

Supplement: FIG S2 [file mbio.02781-21-sf002.tif]

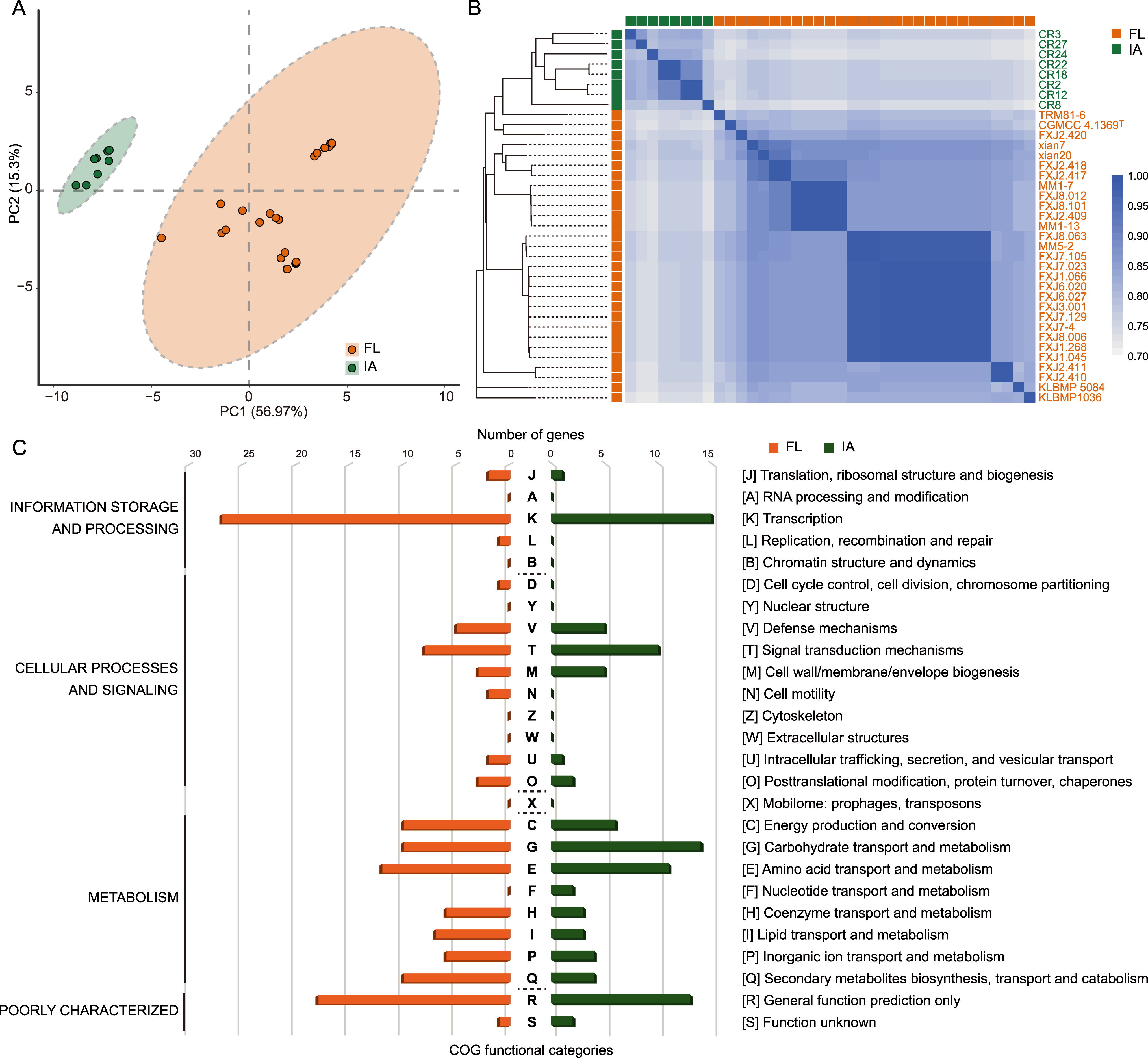

Supplement: FIG S3 [file mbio.02781-21-sf003.tif]

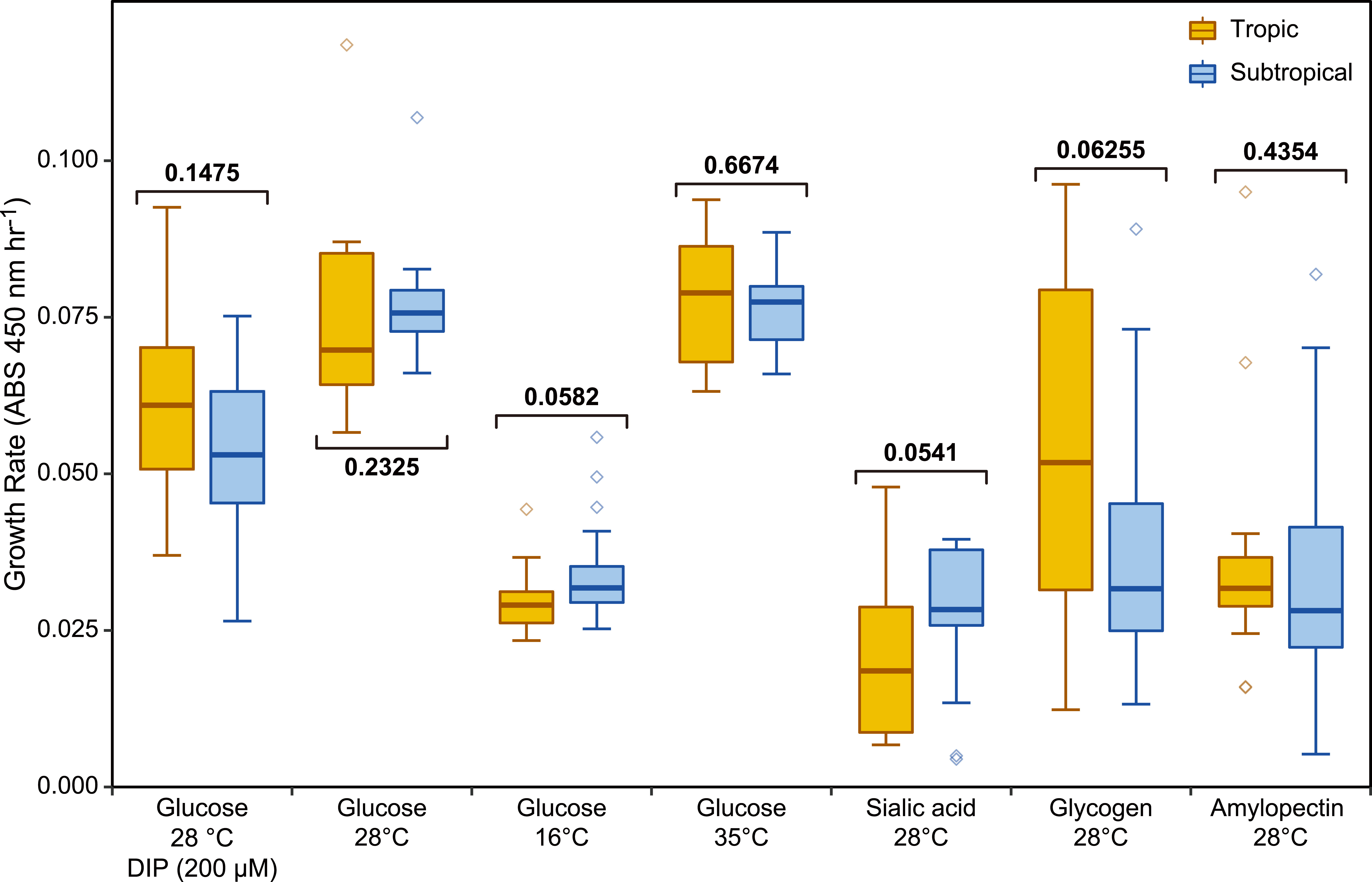

Supplement: FIG S4 [file mbio.02781-21-sf004.tif]

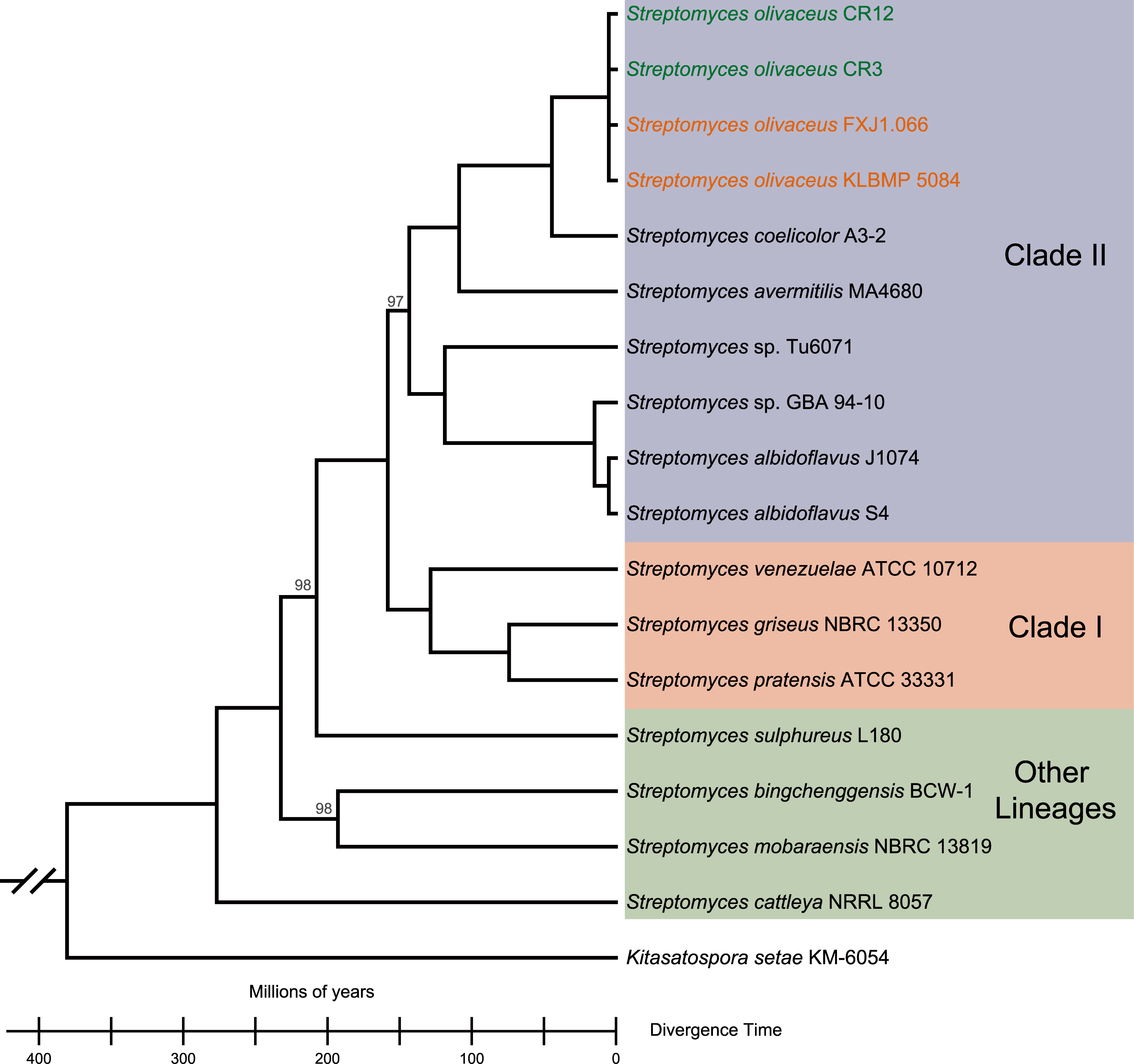

Supplement: FIG S5 [file mbio.02781-21-sf005.tif]
