# Supplementary material for: Habitat Adaptation Drives Speciation of a Streptomyces Species with Distinct Habitats and Disparate Geographic Origins
Source: mBio. 2022 Jan 11;13(1):e02781-21. doi: 10.1128/mbio.02781-21 (PMC8749437; doi:10.1128/mbio.02781-21)
Supplement: TABLE S1 [file mbio.02781-21-st001.docx]

Table S1. Genomic and geographic characteristics of *S. olivaceus* strains used in this study.

| **Strain** | **No. of contig** | **Contig_N50 Length (bp)** | **Genome size (Mb)** | **ANI (%) to KLBMP 5084** | **No. of Coding**  **Sequence** | **Clade*^a^*** | **Origin (geographic location)** | **Habitat*^b^*** | **GenBank accession** | **Reference** |
| --- | --- | --- | --- | --- | --- | --- | --- | --- | --- | --- |
| CR2 | 58 | 370,611 | 8.25 | 97.52 | 7,373 | IA | Guanacaste Conservation Area, Costa Rica (10°51′N, 85°37′W) | Imperial moth (adult body of EI) | JAHSSI000000000 | ([1](#_ENREF_1)) |
| CR3 | 41 | 552,387 | 8.39 | 97.39 | 7,154 | IA | Guanacaste Conservation Area, Costa Rica (10°51′N, 85°37′W) | Imperial moth (adult wing of EI) | JAHSSJ000000000 | ([1](#_ENREF_1)) |
| CR8 | 46 | 623,166 | 8.31 | 97.51 | 7,043 | IA | Guanacaste Conservation Area, Costa Rica (10°51′N, 85°37′W) | Imperial moth (adult body of EI) | JAHSSK000000000 | ([1](#_ENREF_1)) |
| CR12 | 53 | 396,632 | 8.26 | 97.50 | 7,127 | IA | Guanacaste Conservation Area, Costa Rica (10°51′N, 85°37′W) | Imperial moth (adult body of EI) | JAHSSL000000000 | ([1](#_ENREF_1)) |
| CR18 | 65 | 274,188 | 8.25 | 97.47 | 7,165 | IA | Guanacaste Conservation Area, Costa Rica (10°51′N, 85°37′W) | Imperial moth (adult wing of EI) | JAHSSM000000000 | ([1](#_ENREF_1)) |
| CR22 | 52 | 388,264 | 8.26 | 96.93 | 7,132 | IA | Guanacaste Conservation Area, Costa Rica (10°51′N, 85°37′W) | Imperial moth (adult body of EI) | JAHSSN000000000 | ([1](#_ENREF_1)) |
| CR24 | 71 | 362,295 | 8.54 | 97.51 | 7,163 | IA | Guanacaste Conservation Area, Costa Rica (10°51′N, 85°37′W) | Imperial moth (pupa of EI) | JAHSSO000000000 | ([1](#_ENREF_1)) |
| CR27 | 42 | 447,150 | 8.55 | 97.53 | 7,455 | IA | Guanacaste Conservation Area, Costa Rica (10°51′N, 85°37′W) | Imperial moth (pupa inside of EI) | JAHSSP000000000 | ([1](#_ENREF_1)) |
| FXJ1.045 | 66 | 352,379 | 8.26 | 98.92 | 7,134 | FL | Yaoli, Jiangxi Province, China (29°30′N, 117°33′E) | Soil | JAHSSQ000000000 | ([1](#_ENREF_1)) |
| FXJ1.066 | 68 | 272,643 | 8.26 | 98.92 | 7,141 | FL | Nanchang, Jiangxi Province, China (28°45′N, 115°49′E) | Soil | JAHSSR000000000 | ([1](#_ENREF_1)) |
| FXJ1.268 | 60 | 377,601 | 8.26 | 98.93 | 7,130 | FL | Wushan, Jiangxi Province, China (29°44′N, 115°37′E) | Soil | JAHSSS000000000 | ([1](#_ENREF_1)) |
| FXJ2.409 | 63 | 344,713 | 8.37 | 99.04 | 7,252 | FL | Huangshan, Anhui Province, China (30°8′N, 118°10′E) | Soil | JAHSST000000000 | ([1](#_ENREF_1)) |
| FXJ2.410 | 65 | 343,607 | 8.37 | 99.10 | 7,358 | FL | Huangshan, Anhui Province, China (30°8′N, 118°10′E) | Soil | JAHSSU000000000 | ([1](#_ENREF_1)) |
| FXJ2.411 | 66 | 343,431 | 8.33 | 99.09 | 7,332 | FL | Huangshan, Anhui Province, China (30°8′N, 118°10′E) | Soil | JAHSSV000000000 | ([1](#_ENREF_1)) |
| FXJ2.417 | 73 | 295,693 | 8.28 | 99.14 | 7,118 | FL | Kaifeng, Henan Province, China (34°49′N, 114°18′E) | Soil | JAHSSW000000000 | ([1](#_ENREF_1)) |
| FXJ2.418 | 71 | 290,635 | 8.25 | 99.13 | 7,092 | FL | Kaifeng, Henan Province, China (34°49′N, 114°18′E) | Soil | JAHSSX000000000 | ([1](#_ENREF_1)) |
| FXJ2.420 | 51 | 396,789 | 8.33 | 98.63 | 7,232 | FL | Zibo, Shandong Province, China (36°20′N, 118°3′E) | Soil | JAHSSY000000000 | ([1](#_ENREF_1)) |
| FXJ3.001 | 72 | 272,643 | 8.26 | 98.91 | 7,135 | FL | Xishuangbanna, Yunnan Province, China (22°0′N, 100°48′E) | Earthnut (ST) | JAHSSZ000000000 | ([1](#_ENREF_1)) |
| TRM81-6 | 56 | 357,003 | 8.42 | 98.68 | 7,512 | FL | Aqsu, Xinjiang Province, China (40°22′N, 80°3′E) | Farmland soil | JAHSTA000000000 | ([1](#_ENREF_1)) |
| MM1-13 | 55 | 424,850 | 8.37 | 99.04 | 7,293 | FL | Nanchang, Jiangxi Province, China (28°45′N, 115°49′E) | Earthworm | JAHSTB000000000 | ([1](#_ENREF_1)) |
| MM1-7 | 58 | 352,835 | 8.16 | 99.04 | 7,247 | FL | Nanchang, Jiangxi Province, China (28°45′N, 115°49′E) | Earthworm | JAHSTC000000000 | ([1](#_ENREF_1)) |
| MM5-2 | 58 | 405,348 | 8.6 | 98.94 | 7,007 | FL | Nanchang, Jiangxi Province, China (28°45′N, 115°49′E) | Earthworm | JAHSTD000000000 | ([1](#_ENREF_1)) |
| CGMCC 4.1369^T^ | 68 | 337,880 | 8.66 | 98.23 | 7,451 | FL | —*^c^* | Soil | JAHSTE000000000 | ([2](#_ENREF_2)) |
| xian7 | 54 | 437,192 | 8.11 | 99.05 | 6,966 | FL | Danzhou, Hainan Province, China (19°52′N, 109°57′E) | Root knot nematode (*Meloidogyne*) | JAHSTF000000000 | ([1](#_ENREF_1)) |
| xian20 | 59 | 391,164 | 8.12 | 99.01 | 6,973 | FL | Sanya, Hainan Province, China (18°25′N, 109°49′E) | Root knot nematode (*Meloidogyne*) | JAHSTG000000000 | ([1](#_ENREF_1)) |
| KLBMP 5084 | 1 | — | 8.18 | — | 7,250 | FL | Jiangsu Province, China (32°19′N, 118°30′E) | plant tissues | NZ_CP016795.1 | ([3](#_ENREF_3)) |
| KLBMP 1036 | 61 | 292,353 | 8.36 | 99.17 | 7,459 | FL | Panxi plateau in South-west Sichuan, China (26°58′N, 102°12′E) | plant tissues | JAHSTH000000000 | ([4](#_ENREF_4)) |
| FXJ6.020 | 64 | 320,192 | 8.34 | 98.94 | 7,210 | FL | South China Sea (19°39′N, 109°04′E, depth, 7-10 m) | Sponge (*Xestosponiga* sp. QYP07) | JAHSTI000000000 | ([1](#_ENREF_1)) |
| FXJ6.027 | 60 | 320,298 | 8.26 | 98.91 | 7,134 | FL | South China Sea (18°19′N, 108°57′E, depth, 7-10 m) | Sponge (*Xestospongia* sp. SYM12) | JAHSTJ000000000 | ([1](#_ENREF_1)) |
| FXJ7.023 | 67 | 254,377 | 8.26 | 98.92 | 7,129 | FL | South China Sea (19°53′N, 115°6′E, depth, 1,182 m) | Deep-sea sediment | JAHSTK000000000 | ([5](#_ENREF_5)) |
| FXJ7.105 | 64 | 352,836 | 8.25 | 98.91 | 7,089 | FL | South China Sea (19°53′N, 115°6′E, depth, 1,182 m) | Deep-sea sediment | JAHSTL000000000 | ([1](#_ENREF_1)) |
| FXJ7.129 | 66 | 272,621 | 8.27 | 98.94 | 7,139 | FL | Yellow Sea (39°1′N, 121°46′E, depth, < 1 m) | Coastal sediment | JAHSTM000000000 | ([1](#_ENREF_1)) |
| FXJ7-4 | 64 | 352,039 | 8.29 | 98.91 | 7,152 | FL | South China Sea (17°33′N, 110°22′E, depth, 3,000 m) | Deep-sea sediment | JAHSTN000000000 | ([1](#_ENREF_1)) |
| FXJ8.006 | 68 | 313,388 | 8.26 | 98.93 | 7,134 | FL | Southern Indian Ocean (37°49′S, 62°00′E, depth, 3,500 m) | Deep-sea water | JAHSTO000000000 | ([1](#_ENREF_1)) |
| FXJ8.012 | 57 | 424,850 | 8.37 | 99.04 | 7,245 | FL | Southern Indian Ocean (37°49′S, 72°00′E, depth, 3,838 m) | Deep-sea water | JAHSTP000000000 | ([6](#_ENREF_6)) |
| FXJ8.063 | 60 | 326,506 | 8.16 | 98.95 | 7,009 | FL | Southwest Indian Ocean (37°49'S, 49°37'E, depth, 2,800 m) | Deep-sea sediment | JAHSTQ000000000 | ([1](#_ENREF_1)) |
| FXJ8.101 | 63 | 355,376 | 8.42 | 99.04 | 7,293 | FL | Southwest India Ocean (38°57′S, 47°24′E, depth, 1,880 m) | Deep-sea sediment | JAHSTR000000000 | ([1](#_ENREF_1)) |

*^a^* IA, insect-associated; FL, free-living.

*^b^* EI, *Eacles imperialis*; ST, *Stemona tuberos*a. Homogenates of the rinsed samples, i.e., pupa, adult body, adult wing and leaf, were used for isolation.

*^c^* -, Information unavailable.

References

1. Cheng K. 2016. The roles of homologous recombination and habitat barriers to it in the evolution of streptomycetes. PhD thesis. University of Chinese Academy of Sciences, Beijing.

2. Skerman VBD, McGowan V, Sneath PHA. 1980. Approved lists of bacterial names. Int J Syst Bacteriol 30:225-420.

3. Qin S, Feng W-W, Wang T-T, Ding P, Xing K, Jiang J-H. 2017. Plant growth-promoting effect and genomic analysis of the beneficial endophyte *Streptomyces* sp. KLBMP 5084 isolated from halophyte *Limonium sinense*. Plant Soil 416:117-132.

4. Qin S, Miao Q, Feng W-W, Wang Y, Zhu X, Xing K, Jiang J-H. 2015. Biodiversity and plant growth promoting traits of culturable endophytic actinobacteria associated with *Jatropha curcas* L. growing in Panxi dry-hot valley soil. Appl Soil Ecol 93:47-55.

5. Yue C, Liu N, Liu M, Lu Y, Shao M, Wang M, Ai G, Huang Y. 2015. Tandem expression in *E. coli* of type III PKS and P450 genes from marine *Streptomyces olivaceus* FXJ 7.023 gives production of phenol and indole. World J Microbiol Biotechnol 31:541-548.

6. Liu N, Song F, Shang F, Huang Y. 2015. Mycemycins A-E, new dibenzoxazepinones isolated from two different streptomycetes. Mar Drugs 13:6247-6258.
