# Supplementary material for: Habitat Adaptation Drives Speciation of a Streptomyces Species with Distinct Habitats and Disparate Geographic Origins
Source: mBio. 2022 Jan 11;13(1):e02781-21. doi: 10.1128/mbio.02781-21 (PMC8749437; doi:10.1128/mbio.02781-21)
Supplement: TABLE S2 [file mbio.02781-21-st002.docx]

Table S2. The core secondary metabolite biosynthetic gene clusters of *S. olivaceus* predicted by antiSMASH 5.0.

| **Predicted biosynthetic system** | **Position start (bp)*^a^*** | **Position end (bp)*^a^*** | **Predicted product** |
| --- | --- | --- | --- |
| Hybrid NRPS / PKS | 21,315 | 126,113 | Totopensamide |
| Terpene | 138,469 | 158,810 | Unknown |
| Type III PKS | 181,209 | 222,301 | Germicidin |
| Indole | 367,311 | 388,438 | Unknown |
| Terpene | 439,866 | 462,090 | Carotenoid |
| Amglyccycl | 645,474 | 666,480 | Unknown |
| Type III PKS | 899,758 | 940,870 | Unknown |
| NRPS | 1,013,786 | 1,065,451 | Unknown |
| Ectoine | 1,590,268 | 1,600,666 | Ectoine |
| Melanin | 2,629,590 | 2,640,216 | Melanin |
| Lanthipeptide | 4,175,894 | 4,204,080 | SBI 06990 alpha / SBI 06989 beta |
| NRPS | 4,591,549 | 4,652,308 | Unknown |
| Terpene | 5,348,767 | 5,368,585 | Albaflavenone |
| Type II PKS | 5,419,057 | 5,491,611 | Spore pigment |
| Siderophore | 5,973,602 | 5,983,835 | Aerobactin-like |
| Hybrid NRPS / PKS | 6,056,055 | 6,161,799 | Unknown |
| Hybrid NRPS / PKS | 6,206,918 | 6,254,735 | Xiamycin |
| Bacteriocin | 6,290,809 | 6,301,651 | Unknown |
| Terpene | 6,331,530 | 6,352,760 | Geosmin |
| Siderophore | 6,500,626 | 6,513,800 | Unknown |
| NRPS | 6,550,791 | 6,596,156 | Diisonitrile antibiotic SF2768 |
| Terpene | 6,987,115 | 7,060,211 | Hopene |
| Hybrid NRPS / PKS | 7,081,623 | 7,222,660 | Divergolide |
| Terpene | 7,522,335 | 7,543,363 | Unknown |
| Bacteriocin | 7,552,695 | 7,562,910 | Informatipeptin |
| NRPS | 7,767,319 | 7,818,238 | Coelichelin |
| Type I PKS | 7,955,747 | 8,044,335 | Unknown |

*^a^* Determined by the relative location in the complete genome of *S. olivaceus* KLBMP 5084.
