# Supplementary material for: Habitat Adaptation Drives Speciation of a Streptomyces Species with Distinct Habitats and Disparate Geographic Origins
Source: mBio. 2022 Jan 11;13(1):e02781-21. doi: 10.1128/mbio.02781-21 (PMC8749437; doi:10.1128/mbio.02781-21)
Supplement: TABLE S3 [file mbio.02781-21-st003.docx]

Table S3. Functional annotation of genes in the *S. olivaceus* genomic regions with four times the average clade-specific SNPs.

| **Region** | **Locus ID*^a^*** | **Start (bp)*****^b^*** | **End (bp)*^b^*** | **Length (bp)** | **No. of SNPs** | **No. of clade-specific SNPs** | **COG number/family** | **COG category** | **Function annotation** |
| --- | --- | --- | --- | --- | --- | --- | --- | --- | --- |
| 1 | KLBMP 5084_1726 | 1,231,736 | 1,233,522 | 1785 | 116 | 32 | COG1506 | E | Prolyl oligopeptidase family serine peptidase |
| 2 | KLBMP 5084_2059 | 1,463,849 | 1,464,765 | 915 | 69 | 43 | COG0583 | K | LysR family transcriptional regulator |
| 3 | KLBMP 5084_3288 | 2,303,024 | 2,304,339 | 1314 | 74 | 32 | COG4585 | T | Two-component sensor histidine kinase [EC:2.7.13.3] |
| 4 | KLBMP 5084_6574 | 4,504,598 | 4,506,252 | 1653 | 90 | 25 | COG2208 | T; K | SpoIIE family protein phosphatase [EC:3.1.3.3] |

*^a^* Genes are represented by loci from strain KLBMP 5084.

*^b^* Based on the alignment of 5,007 single-copy core genes.
