# Supplementary material for: Habitat Adaptation Drives Speciation of a Streptomyces Species with Distinct Habitats and Disparate Geographic Origins
Source: mBio. 2022 Jan 11;13(1):e02781-21. doi: 10.1128/mbio.02781-21 (PMC8749437; doi:10.1128/mbio.02781-21)
Supplement: TABLE S4 [file mbio.02781-21-st004.docx]

Table S4. Functional annotation of clade-specific genes in *S. olivaceus*.

| **Locus id*^a^*** | **Clade** | **COG number/family** | **COG category** | **Function annotation** |
| --- | --- | --- | --- | --- |
| KLBMP 5084_187 | FL | COG0477 | G; E; P; R | MFS transporter |
| KLBMP 5084_293 | FL | COG0491 | R | MBL fold metallo-hydrolase |
| KLBMP 5084_294 | FL | COG1733 | K | HxlR family transcriptional regulator |
| KLBMP 5084_297 | FL | — | — | hypothetical protein |
| KLBMP 5084_458 | FL | — | — | DUF5133 domain-containing protein |
| KLBMP 5084_509 | FL | COG0590 | J | hypothetical protein |
| KLBMP 5084_605 | FL | COG3010 | G | N-acylglucosamine-6-phosphate 2-epimerase [EC:5.1.3.9] |
| KLBMP 5084_606 | FL | COG1940 | K; G | ROK family protein |
| KLBMP 5084_607 | FL | COG0329 | E; M | dihydrodipicolinate synthase family protein |
| KLBMP 5084_608 | FL | COG4409 | G; M | sialidase-1 [EC:3.2.1.18] |
| KLBMP 5084_609 | FL | COG3458 | Q | cephalosporin-C deacetylase [EC:3.1.1.41] |
| KLBMP 5084_610 | FL | COG2186 | K | FadR family transcriptional regulator |
| KLBMP 5084_611 | FL | COG0747 | E | ABC transporter substrate-binding protein |
| KLBMP 5084_612 | FL | COG0601 | E; P | ABC transporter permease |
| KLBMP 5084_613 | FL | COG0444 | E; P | dipeptide/oligopeptide/nickel ABC transporter permease/ATP-binding protein |
| KLBMP 5084_614 | FL | COG0444 | E; P | dipeptide/oligopeptide/nickel ABC transporter permease/ATP-binding protein |
| KLBMP 5084_615 | FL | COG4608 | E | ABC transporter ATP-binding protein |
| KLBMP 5084_782 | FL | COG2226 | H | class I SAM-dependent methyltransferase |
| KLBMP 5084_783 | FL | COG0334 | E | Glu/Leu/Phe/Val dehydrogenase |
| KLBMP 5084_784 | FL | COG1522 | K | Lrp/AsnC family transcriptional regulator |
| KLBMP 5084_796 | FL | — | — | hypothetical protein |
| KLBMP 5084_864 | FL | COG1131 | V | ABC transporter ATP-binding protein |
| KLBMP 5084_865 | FL | COG4585 | T | two-component sensor histidine kinase |
| KLBMP 5084_866 | FL | COG2197 | T; K | response regulator transcription factor |
| KLBMP 5084_916 | FL | — | — | hypothetical protein |
| KLBMP 5084_917 | FL | COG1846 | K | MarR family transcriptional regulator |
| KLBMP 5084_918 | FL | COG2220 | G | MBL fold metallo-hydrolase |
| KLBMP 5084_919 | FL | COG1846 | K | MarR family transcriptional regulator |
| KLBMP 5084_931 | FL | — | — | hypothetical protein |
| KLBMP 5084_950 | FL | — | — | hypothetical protein BC342_04585 |
| KLBMP 5084_1456 | FL | COG0604 | C; R | alcohol dehydrogenase |
| KLBMP 5084_1457 | FL | COG1917; COG4977 | R; K | AraC family transcriptional regulator |
| KLBMP 5084_1591 | FL | COG0705 | O | rhomboid family intramembrane serine protease |
| KLBMP 5084_1755 | FL | COG0702 | R | NAD(P)H dehydrogenase (quinone) [EC:1.6.5.2] |
| KLBMP 5084_1756 | FL | COG1846 | K | MarR family transcriptional regulator |
| KLBMP 5084_1787 | FL | COG0366 | G | hypothetical protein |
| KLBMP 5084_1855 | FL | COG2172 | T | serine/threonine-protein kinase RsbW [EC:2.7.11.1] |
| KLBMP 5084_1926 | FL | COG2173 | M | dipeptidase |
| KLBMP 5084_1939 | FL | — | — | peptidoglycan-binding protein |
| KLBMP 5084_2043 | FL | — | — | hypothetical protein |
| KLBMP 5084_2058 | FL | COG3227 | O | M4 family peptidase |
| KLBMP 5084_2060 | FL | COG1028 | I; Q; R | SDR family oxidoreductase |
| KLBMP 5084_2093 | FL | — | — | DUF397 domain-containing protein |
| KLBMP 5084_2417 | FL | COG0431 | C | NADPH-dependent oxidoreductase |
| KLBMP 5084_2418 | FL | COG1396 | K | XRE family transcriptional regulator |
| KLBMP 5084_2430 | FL | COG0076 | E | L-2,4-diaminobutyrate decarboxylase [EC:4.1.1.86] |
| KLBMP 5084_2431 | FL | COG3486 | Q | lysine N6-hydroxylase [EC:1.14.13.59] |
| KLBMP 5084_2432 | FL | COG1670 | J; O | N-acetyltransferase |
| KLBMP 5084_2433 | FL | COG4264 | P | desferrioxamine E synthetase monomer |
| KLBMP 5084_2625 | FL | COG0477 | G; E; P; R | MFS transporter |
| KLBMP 5084_2937 | FL | COG1733 | K | transcriptional regulator |
| KLBMP 5084_2938 | FL | COG0604 | C; R | NADPH:quinone reductase [EC:1.6.5.5] |
| KLBMP 5084_2946 | FL | COG1317 | N; U | hypothetical protein |
| KLBMP 5084_3056 | FL | — | — | hypothetical protein |
| KLBMP 5084_3057 | FL | — | — | hypothetical protein |
| KLBMP 5084_3100 | FL | COG2207 | K | helix-turn-helix domain-containing protein |
| KLBMP 5084_3101 | FL | COG2207 | K | helix-turn-helix domain-containing protein |
| KLBMP 5084_3102 | FL | COG4319 | R | SgcJ/EcaC family oxidoreductase |
| KLBMP 5084_3112 | FL | — | — | hypothetical protein |
| KLBMP 5084_3237 | FL | COG4221 | C | SDR family NAD(P)-dependent oxidoreductase |
| KLBMP 5084_3238 | FL | COG2207 | K | helix-turn-helix domain-containing protein |
| KLBMP 5084_3245 | FL | — | — | hypothetical protein |
| KLBMP 5084_3249 | FL | COG2197 | T; K | response regulator transcription factor |
| KLBMP 5084_3250 | FL | COG1465 | E | 3-amino-4-hydroxybenzoic acid synthase [EC:4.1.99.20] |
| KLBMP 5084_3251 | FL | COG1830 | G | 2-amino-4,5-dihydroxy-6-oxo-7-(phosphooxy)heptanoate synthase [EC:4.1.2.56] |
| KLBMP 5084_3252 | FL | COG0318 | I; Q | long-chain fatty acidCoA ligase |
| KLBMP 5084_3253 | FL | COG0778 | C | SagB/ThcOx family dehydrogenase |
| KLBMP 5084_3254 | FL | — | — | thiazolylpeptide-type bacteriocin |
| KLBMP 5084_3255 | FL | — | — | hypothetical protein |
| KLBMP 5084_3256 | FL | — | — | hypothetical protein |
| KLBMP 5084_3257 | FL | — | — | hypothetical protein |
| KLBMP 5084_3258 | FL | — | — | hypothetical protein |
| KLBMP 5084_3259 | FL | — | — | hypothetical protein |
| KLBMP 5084_3260 | FL | — | — | hypothetical protein |
| KLBMP 5084_3261 | FL | — | — | hypothetical protein |
| KLBMP 5084_3262 | FL | — | — | TOMM precursor leader peptide-binding protein |
| KLBMP 5084_3263 | FL | — | — | hypothetical protein |
| KLBMP 5084_3264 | FL | — | — | hypothetical protein |
| KLBMP 5084_3265 | FL | — | — | hypothetical protein |
| KLBMP 5084_3266 | FL | COG0654 | H; C | salicylate hydroxylase [EC:1.14.13.1] |
| KLBMP 5084_3267 | FL | COG0654 | H; C | salicylate hydroxylase [EC:1.14.13.1] |
| KLBMP 5084_3268 | FL | COG0842 | V | ABC transporter permease |
| KLBMP 5084_3269 | FL | COG1131 | V | ABC transporter ATP-binding protein |
| KLBMP 5084_3514 | FL | — | — | hypothetical protein |
| KLBMP 5084_3569 | FL | COG3509 | Q | hypothetical protein |
| KLBMP 5084_3645 | FL | COG3239 | I | acyl-CoA desaturase |
| KLBMP 5084_3646 | FL | — | — | hypothetical protein |
| KLBMP 5084_3647 | FL | COG2203; COG2208 | T; K | phosphoserine phosphatase RsbU/P [EC:3.1.3.3] |
| KLBMP 5084_3719 | FL | COG1826 | U | Sec-independent protein translocase subunit TatA |
| KLBMP 5084_3876 | FL | — | — | nuclear transport factor 2 family protein |
| KLBMP 5084_3878 | FL | COG1028 | I; Q; R | glucose 1-dehydrogenase |
| KLBMP 5084_3881 | FL | COG0667 | R | 1-deoxyxylulose-5-phosphate synthase [EC:1.1.-.-] |
| KLBMP 5084_3883 | FL | COG2378 | K | YafY family transcriptional regulator |
| KLBMP 5084_3884 | FL | COG1694 | V | hypothetical protein |
| KLBMP 5084_3886 | FL | — | — | hypothetical protein |
| KLBMP 5084_3888 | FL | COG1396 | K | XRE family transcriptional regulator |
| KLBMP 5084_3889 | FL | COG3268 | S | saccharopine dehydrogenase |
| KLBMP 5084_3945 | FL | COG1846 | K | MarR family transcriptional regulator |
| KLBMP 5084_3946 | FL | COG0596 | H; R | alpha/beta hydrolase |
| KLBMP 5084_3947 | FL | — | — | hypothetical protein |
| KLBMP 5084_3948 | FL | COG1476 | K | XRE family transcriptional regulator |
| KLBMP 5084_3949 | FL | — | — | DUF397 domain-containing protein |
| KLBMP 5084_3968 | FL | — | — | hypothetical protein |
| KLBMP 5084_4047 | FL | — | — | XRE family transcriptional regulator |
| KLBMP 5084_4070 | FL | — | — | hypothetical protein EV562_10638 |
| KLBMP 5084_4257 | FL | COG0852 | C | dehydrogenase |
| KLBMP 5084_4318 | FL | COG5464 | L | hypothetical protein |
| KLBMP 5084_4508 | FL | — | — | hypothetical protein |
| KLBMP 5084_4582 | FL | COG2220 | G | MBL fold metallo-hydrolase |
| KLBMP 5084_4583 | FL | COG1309 | K | TetR/AcrR family transcriptional regulator |
| KLBMP 5084_4588 | FL | — | — | hypothetical protein |
| KLBMP 5084_4589 | FL | — | — | hypothetical protein |
| KLBMP 5084_4615 | FL | — | — | hypothetical protein |
| KLBMP 5084_4637 | FL | — | — | hypothetical protein |
| KLBMP 5084_4731 | FL | — | — | hypothetical protein |
| KLBMP 5084_5289 | FL | — | — | hypothetical protein |
| KLBMP 5084_5304 | FL | COG0455 | D; N | MinD/ParA family protein |
| KLBMP 5084_5313 | FL | — | — | thymidylate synthase (FAD) [EC:2.1.1.148] |
| KLBMP 5084_5410 | FL | COG1309 | K | TetR/AcrR family transcriptional regulator |
| KLBMP 5084_5411 | FL | COG2249 | R | oxidoreductase |
| KLBMP 5084_5416 | FL | COG1246 | E | putative acetyltransferase [EC:2.3.1.-] |
| KLBMP 5084_5417 | FL | COG1028 | I; Q; R | SDR family oxidoreductase |
| KLBMP 5084_5419 | FL | COG0702 | R | SDR family oxidoreductase |
| KLBMP 5084_5437 | FL | — | — | hypothetical protein |
| KLBMP 5084_5460 | FL | COG4585 | T | two-component sensor histidine kinase |
| KLBMP 5084_5461 | FL | COG2197 | T; K | response regulator transcription factor |
| KLBMP 5084_5462 | FL | COG1680 | V | D-alanyl-D-alanine carboxypeptidase [EC:3.4.16.4] |
| KLBMP 5084_5471 | FL | COG2197 | T; K | LuxR family transcriptional regulator |
| KLBMP 5084_5472 | FL | — | — | hypothetical protein |
| KLBMP 5084_5473 | FL | COG1028 | I; Q; R | 3-oxoacyl-[acyl-carrier protein] reductase [EC:1.1.1.100] |
| KLBMP 5084_5479 | FL | COG1233 | Q | NAD(P)/FAD-dependent oxidoreductase |
| KLBMP 5084_5480 | FL | COG1309 | K | TetR family transcriptional regulator |
| KLBMP 5084_5481 | FL | COG0654 | H; C | oxidoreductase |
| KLBMP 5084_5482 | FL | COG0654 | H; C | oxidoreductase |
| KLBMP 5084_5483 | FL | — | — | hypothetical protein |
| KLBMP 5084_5518 | FL | COG2130 | Q; R | NADP-dependent oxidoreductase |
| KLBMP 5084_5520 | FL | COG4977 | K | helix-turn-helix domain-containing protein |
| KLBMP 5084_5521 | FL | COG0662 | G | cupin domain-containing protein |
| KLBMP 5084_5522 | FL | COG1062 | R | aryl-alcohol dehydrogenase [EC:1.1.1.90] |
| KLBMP 5084_5523 | FL | COG0583 | K | LysR family transcriptional regulator |
| KLBMP 5084_5760 | FL | — | — | hypothetical protein BC342_28035 |
| KLBMP 5084_5892 | FL | — | — | hypothetical protein |
| KLBMP 5084_6073 | FL | — | — | hypothetical protein BC342_29525 |
| KLBMP 5084_6082 | FL | — | — | nitrate reductase subunit alpha |
| KLBMP 5084_6315 | FL | — | — | sensor histidine kinase |
| KLBMP 5084_6335 | FL | — | — | hypothetical protein |
| KLBMP 5084_6739 | FL | — | — | — |
| KLBMP 5084_6811 | FL | — | — | hypothetical protein |
| KLBMP 5084_7114 | FL | — | — | hypothetical protein |
| KLBMP 5084_7115 | FL | COG2230 | I | methyltransferase domain-containing protein |
| CR12_85 | IA | COG1917; COG2207 | R; K | AraC family transcriptional regulator |
| CR12_86 | IA | COG1120 | P; H | ferric hydroxamate transport system ATP-binding protein [EC:7.2.2.16] |
| CR12_87 | IA | COG0614 | P | ferric hydroxamate transport system substrate-binding protein |
| CR12_88 | IA | COG0609 | P | ferric hydroxamate transport system permease protein |
| CR12_143 | IA | COG3387 | G | alpha-L-rhamnosidase [EC:3.2.1.40] |
| CR12_144 | IA | COG3250 | G | twin-arginine translocation signal domain-containing protein |
| CR12_145 | IA | COG3533 | S | glycosyl hydrolase of unknown function (DUF1680) |
| CR12_262 | IA | COG1309 | K | TetR/AcrR family transcriptional regulator |
| CR12_269 | IA | COG2755 | E | SGNH/GDSL hydrolase family protein |
| CR12_270 | IA | COG1835 | M | acyltransferase |
| CR12_331 | IA | COG1787 | V | restriction endonuclease |
| CR12_448 | IA | COG2170 | O | glutamate---cysteine ligase / carboxylate-amine ligase [EC:6.3.2.2 6.3.-.-] |
| CR12_514 | IA | — | — | MULTISPECIES: membrane protein |
| CR12_568 | IA | COG0329 | E; M | dihydrodipicolinate synthase family protein |
| CR12_569 | IA | COG4948 | M; R | galactonate dehydratase [EC:4.2.1.6] |
| CR12_837 | IA | COG0531 | E | amino acid permease |
| CR12_938 | IA | COG2267 | I | alpha/beta hydrolase |
| CR12_939 | IA | COG2208 | T; K | phosphoserine phosphatase RsbU/P [EC:3.1.3.3] |
| CR12_940 | IA | COG1366 | T | anti-sigma factor antagonist |
| CR12_1490 | IA | COG0705 | O | OSC60795.1 rhomboid family intramembrane serine protease |
| CR12_1679 | IA | COG0366 | G | pullulanase [EC:3.2.1.41] |
| CR12_1884 | IA | — | — | hypothetical protein |
| CR12_1938 | IA | — | — | DUF4190 domain-containing protein |
| CR12_1988 | IA | — | — | DUF397 domain-containing protein |
| CR12_2033 | IA | COG0451 | M | SDR family oxidoreductase |
| CR12_2034 | IA | COG1309 | K | TetR/AcrR family transcriptional regulator |
| CR12_2239 | IA | COG0028 | E; H | thiamine pyrophosphate-binding protein |
| CR12_2405 | IA | COG2814 | G | MFS transporter |
| CR12_2406 | IA | COG1917; COG2207 | R; K | AraC family transcriptional regulator |
| CR12_2434 | IA | COG0477 | G; E; P; R | MFS transporter |
| CR12_2485 | IA | COG2211 | G | MFS transporter |
| CR12_2972 | IA | — | — | hypothetical protein |
| CR12_3182 | IA | — | — | hypothetical protein |
| CR12_3425 | IA | — | — | hypothetical protein |
| CR12_3468 | IA | COG2208 | T; K | serine/threonine-protein phosphatase |
| CR12_3476 | IA | COG3509 | Q | hypothetical protein |
| CR12_3623 | IA | COG1826 | U | Sec-independent protein translocase subunit TatA |
| CR12_3844 | IA | COG5635 | T | NACHT domain-containing protein |
| CR12_3845 | IA | COG5635 |  | NACHT domain-containing protein |
| CR12_3937 | IA | COG0583 | K | LysR family transcriptional regulator |
| CR12_4097 | IA | COG0852 | C | dehydrogenase |
| CR12_4346 | IA | COG1309 | K | TetR family transcriptional regulator |
| CR12_4347 | IA | COG0110 | R | virginiamycin A acetyltransferase [EC:2.3.1.-] |
| CR12_4418 | IA | COG3300 | T | hypothetical protein |
| CR12_4423 | IA | COG1787; COG2310 | V; T | restriction endonuclease |
| CR12_5129 | IA | COG1351 | F | thymidylate synthase (FAD) [EC:2.1.1.148] |
| CR12_5271 | IA | COG0515 | T | serine/threonine protein kinase |
| CR12_5272 | IA | — | — | SapB/AmfS family lantipeptide |
| CR12_5273 | IA | COG1132 | V | ABC transporter ATP-binding protein |
| CR12_5274 | IA | COG1132 | V | ABC transporter ATP-binding protein |
| CR12_5275 | IA | COG2197 | T; K | response regulator transcription factor |
| CR12_5277 | IA | COG0583 | K | LysR family transcriptional regulator |
| CR12_5278 | IA | COG3340 | E | dipeptidase E [EC:3.4.13.21] |
| CR12_5279 | IA | COG1414 | K | IclR family transcriptional regulator |
| CR12_5281 | IA | COG1297 | S | OPT family oligopeptide transporter |
| CR12_5282 | IA | COG4126 | E | AroM family protein |
| CR12_5283 | IA | COG4126 | E | allantoin racemase [EC:5.1.99.3] |
| CR12_5284 | IA | COG2124 | Q; V | cytochrome P450 |
| CR12_5820 | IA | COG4952 | M | L-rhamnose isomerase / sugar isomerase [EC:5.3.1.14 5.3.1.-] |
| CR12_5821 | IA | COG3959 | G | transketolase [EC:2.2.1.1] |
| CR12_5822 | IA | COG3958 | G | transketolase family protein |
| CR12_5823 | IA | COG0554 | C | glycerol kinase |
| CR12_5876 | IA | — | — | hypothetical protein |
| CR12_6175 | IA | — | — | hypothetical protein |
| CR12_6176 | IA | — | — | hypothetical protein |
| CR12_6253 | IA | COG1396 | K | XRE family transcriptional regulator |
| CR12_6254 | IA | COG0693 | R | type 1 glutamine amidotransferase domain-containing protein |
| CR12_6431 | IA | COG3622 | G | hydroxypyruvate isomerase [EC:5.3.1.22] |
| CR12_6432 | IA | COG2610 | G; R | gluconate transporter |
| CR12_6433 | IA | COG0473 | C; E | isocitrate/isopropylmalate dehydrogenase family protein |
| CR12_6434 | IA | COG1414 | K | IclR family transcriptional regulator |
| CR12_6435 | IA | COG0129 | E; G | dihydroxy-acid dehydratase |
| CR12_6436 | IA | COG4221 | C | SDR family oxidoreductase |
| CR12_6437 | IA | COG4569 | Q | acetaldehyde dehydrogenase [EC:1.2.1.10] |
| CR12_6438 | IA | COG0119 | E | 4-hydroxy 2-oxovalerate aldolase [EC:4.1.3.39] |
| CR12_6439 | IA | COG0385 | R | bile acid:sodium symporter family protein |
| CR12_6440 | IA | COG0491 | R | N-acyl homoserine lactone hydrolase [EC:3.1.1.81] |
| CR12_6441 | IA | COG1028 | I; Q; R | SDR family oxidoreductase |
| CR12_6442 | IA | — | — | hypothetical protein |
| CR12_6444 | IA | COG1457 | F | cytosine permease |
| CR12_6445 | IA | COG1853; COG3631 | C; R | cag pathogenicity island protein |
| CR12_6446 | IA | — | — | amino acid synthesis family protein |
| CR12_6447 | IA | — | — | amino acid synthesis family protein |
| CR12_6448 | IA | COG2267 | I | (E)-2-((N-methylformamido)methylene)succinate hydrolase [EC:3.5.1.-] |
| CR12_6449 | IA | COG1012 | C | (Z)-2-((N-methylformamido)methylene)-5-hydroxybutyrolactone dehydrogenase [EC:1.2.1.-] |
| CR12_6451 | IA | COG2141 | H; R | flavin-dependent trigonelline monooxygenase, oxygenase component [EC:1.14.14.-] |
| CR12_6452 | IA | COG1802 | K | GntR family transcriptional regulator |
| CR12_6502 | IA | COG1082 | G | glycoside hydrolase family 97 protein |
| CR12_6547 | IA | COG2120 | G | PIG-L family deacetylase |
| CR12_6956 | IA | COG1414 | K | IclR family transcriptional regulator |
| CR12_6957 | IA | COG3964 | R | amidohydrolase/deacetylase family metallohydrolase |
| CR12_6958 | IA | — | — | hypothetical protein |
| CR12_6959 | IA | — | — | hypothetical protein |
| CR12_6960 | IA | — | — | DUF4312 family protein |
| CR12_6961 | IA | — | — | DUF4311 domain-containing protein |
| CR12_6962 | IA | — | — | DUF4310 family protein |
| CR12_6963 | IA | COG1921 | J | D-glucosaminate-6-phosphate ammonia-lyase [EC:4.3.1.29] |
| CR12_6964 | IA | COG1925 | T; G | HPr family phosphocarrier protein |

*^a^* Clades FL and IA-specific genes are represented by loci from strains KLBMP 5084 and CR12, respectively.
