# Supplementary material for: Habitat Adaptation Drives Speciation of a Streptomyces Species with Distinct Habitats and Disparate Geographic Origins
Source: mBio. 2022 Jan 11;13(1):e02781-21. doi: 10.1128/mbio.02781-21 (PMC8749437; doi:10.1128/mbio.02781-21)
Supplement: TABLE S5 [file mbio.02781-21-st005.docx]

Table S5. Growth rates (ABS 450 nm hr^-1^) for *S. olivaceus* under different iron availability, temperature, and sole carbon source conditions.

| **Strain** | **Clade** | **Group** | **Glucose 28 °C DIP***^a^* **(200 μM)** | **Glucose 28 °C** | **Glucose 16 °C** | **Glucose 35 °C** | **Sialic acid 28 °C** | **Glycogen 28 °C** | **Amylopectin 28 °C** |
| --- | --- | --- | --- | --- | --- | --- | --- | --- | --- |
| CR12 | IA | Tropical | 0.070 | 0.068 | 0.037 | 0.065 | 0.008 | 0.034 | 0.033 |
| CR18 | IA | Tropical | 0.067 | 0.066 | 0.031 | 0.063 | 0.009 | 0.041 | 0.030 |
| CR2 | IA | Tropical | 0.071 | 0.057 | 0.024 | 0.085 | 0.008 | 0.074 | 0.034 |
| CR22 | IA | Tropical | 0.060 | 0.060 | 0.023 | 0.083 | 0.009 | 0.050 | 0.036 |
| CR24 | IA | Tropical | 0.093 | 0.118 | 0.031 | 0.094 | 0.012 | 0.093 | 0.095 |
| CR27 | IA | Tropical | 0.084 | 0.075 | 0.031 | 0.081 | 0.008 | 0.032 | 0.030 |
| CR3 | IA | Tropical | 0.079 | 0.063 | 0.027 | 0.068 | 0.010 | 0.090 | 0.024 |
| CR8 | IA | Tropical | 0.050 | 0.087 | 0.026 | 0.072 | 0.007 | 0.062 | 0.032 |
| FXJ1.045 | FL | Subtropical | 0.026 | 0.083 | 0.034 | 0.067 | 0.038 | 0.019 | 0.005 |
| FXJ1.066 | FL | Subtropical | 0.051 | 0.076 | 0.030 | 0.089 | 0.038 | 0.089 | 0.082 |
| FXJ1.268 | FL | Subtropical | 0.037 | 0.080 | 0.025 | 0.077 | 0.040 | 0.051 | 0.066 |
| FXJ2.409 | FL | Subtropical | 0.063 | 0.077 | 0.032 | 0.071 | 0.030 | 0.025 | 0.026 |
| FXJ2.410 | FL | Subtropical | 0.030 | 0.072 | 0.034 | 0.079 | 0.004 | 0.046 | 0.057 |
| FXJ2.411 | FL | Subtropical | 0.055 | 0.075 | 0.050 | 0.082 | 0.005 | 0.024 | 0.025 |
| FXJ2.417 | FL | Subtropical | 0.050 | 0.073 | 0.031 | 0.069 | 0.027 | 0.013 | 0.018 |
| FXJ2.418 | FL | Subtropical | 0.050 | 0.066 | 0.045 | 0.066 | 0.026 | 0.018 | 0.020 |
| FXJ2.420 | FL | Subtropical | 0.057 | 0.077 | 0.038 | 0.074 | 0.040 | 0.019 | 0.024 |
| FXJ3.001 | FL | Tropical | 0.051 | 0.070 | 0.029 | 0.086 | 0.027 | 0.031 | 0.027 |
| FXJ6.020 | FL | Tropical | 0.037 | 0.064 | 0.028 | 0.076 | 0.048 | 0.026 | 0.068 |
| FXJ6.027 | FL | Tropical | 0.052 | 0.069 | 0.026 | 0.087 | 0.032 | 0.087 | 0.032 |
| FXJ7.023 | FL | Tropical | 0.056 | 0.085 | 0.029 | 0.093 | 0.028 | 0.096 | 0.040 |
| FXJ7.105 | FL | Tropical | 0.049 | 0.085 | 0.031 | 0.087 | 0.025 | 0.054 | 0.038 |
| FXJ7.129 | FL | Subtropical | 0.057 | 0.073 | 0.041 | 0.086 | 0.034 | 0.073 | 0.070 |
| FXJ7-4 | FL | Tropical | 0.041 | 0.085 | 0.024 | 0.068 | 0.026 | 0.077 | 0.032 |
| FXJ8.006 | FL | Subtropical | 0.039 | 0.078 | 0.030 | 0.078 | 0.028 | 0.045 | 0.041 |
| FXJ8.012 | FL | Subtropical | 0.072 | 0.079 | 0.029 | 0.076 | 0.026 | 0.032 | 0.030 |
| FXJ8.063 | FL | Subtropical | 0.041 | 0.081 | 0.031 | 0.084 | 0.027 | 0.027 | 0.018 |
| FXJ8.101 | FL | Subtropical | 0.056 | 0.107 | 0.056 | 0.079 | 0.039 | 0.029 | 0.030 |
| KLBMP 1036 | FL | Subtropical | 0.075 | 0.073 | 0.028 | 0.075 | 0.013 | 0.032 | 0.029 |
| KLBMP 5084 | FL | Subtropical | 0.050 | 0.071 | 0.025 | 0.070 | 0.028 | 0.044 | 0.019 |
| MM1-13 | FL | Subtropical | 0.065 | 0.080 | 0.032 | 0.078 | 0.028 | 0.032 | 0.028 |
| MM1-7 | FL | Subtropical | 0.065 | 0.076 | 0.029 | 0.079 | 0.024 | 0.038 | 0.037 |
| MM5-2 | FL | Subtropical | 0.047 | 0.071 | 0.033 | 0.084 | 0.029 | 0.059 | 0.042 |
| TRM81-6 | FL | Subtropical | 0.068 | 0.072 | 0.030 | 0.072 | 0.038 | 0.026 | 0.023 |
| xian20 | FL | Tropical | 0.062 | 0.078 | 0.044 | 0.064 | 0.030 | 0.018 | 0.016 |
| xian7 | FL | Tropical | 0.062 | 0.064 | 0.035 | 0.077 | 0.031 | 0.012 | 0.016 |
| CGMCC 4.1369^T^ | FL | Subtropical | 0.038 | 0.071 | 0.032 | 0.063 | 0.008 | 0.042 | 0.021 |

*^a^* Free iron-limited condition was simulated by adding the iron chelator 2,2’-bipyridyl (DIP).
